# LMNA-PRKDC axis enhances DNA repair and promotes chemoresistance in glioblastoma

**DOI:** 10.1038/s41419-025-08226-3

**Published:** 2025-11-21

**Authors:** Miranda R. Saathoff, Rafal Chojak, Rebecca X. Chen, Hasaan A. Kazi, Umme H. Faisal, Jack M. Shireman, Noah Drewes, Cheol H. Park, Xuesong Fan, Sana A. Khan, Irene Lazanyi, Shivani Baisiwala, C. David James, Craig M. Horbinski, Atique U. Ahmed

**Affiliations:** Department of Neurological Surgery, Feinberg School of Medicine, Northwestern University, Chicago, IL USA

**Keywords:** CNS cancer, Cell signalling

## Abstract

Glioblastoma (GBM) remains one of the deadliest primary brain tumors, with rapid recurrence and near-universal resistance to temozolomide (TMZ) limiting long-term survival. In this study, we identify a clinically actionable mechanism of resistance driven by the *LMNA*–*PRKDC* axis, which enhances DNA repair and tumor cell survival following TMZ treatment. Using patient-derived xenograft models of recurrent GBM, we demonstrate that resistant tumors exhibit elevated *LMNA* expression and increased physical interaction with *PRKDC*, a central regulator of non-homologous end joining (NHEJ). This interaction accelerates the repair of TMZ-induced DNA lesions, contributing to therapeutic failure. Proteomic profiling and targeted immunoprecipitation revealed a distinct *LMNA*–*PRKDC*–associated DNA repair complex. Inhibition of *PRKDC* with the ATP-competitive inhibitor KU57788 reversed resistance, restoring TMZ sensitivity and impairing tumor growth in vivo. Single-cell RNA sequencing of primary and recurrent GBM specimens further identified *LMNA*–*PRKDC* co-expression as a hallmark of treatment-resistant, glioma stem-like cell populations. Importantly, high *LMNA*–*PRKDC* expression was associated with inferior survival outcomes in GBM patient cohorts. These results establish the *LMNA*–*PRKDC* axis as a functional driver of TMZ resistance through enhanced DNA repair capacity in stem-like tumor subpopulations. Our findings support pharmacologic inhibition of *PRKDC* as a rational strategy to resensitize resistant GBM to standard chemotherapy and offer a foundation for future biomarker-driven clinical trials targeting DNA repair vulnerabilities in recurrent disease.

## Introduction

Glioblastoma (GBM) is the most common and aggressive adult primary brain tumor. Current standard-of-care treatment includes maximal safe surgical resection, followed by radiation, temozolomide (TMZ)-based chemotherapy, and tumor-treating fields. Despite this multimodal approach, the prognosis is poor, with a median survival of 14–16 months after diagnosis [1–3].

TMZ, an alkylating agent, exerts cytotoxic effects by inducing DNA double-strand breaks (DSBs), inducing cellular senescence or apoptosis [4]. Although TMZ often produces an initial response, glioblastoma almost invariably recurs with therapy-refractory disease.

Several factors, including MGMT promoter methylation [5, 6], glioma stem cell populations [7], and metabolic adaptation, contribute to TMZ resistance [8]. However, despite extensive research into resistance in recurrent GBM, the mechanisms preventing apoptosis remain elusive. Previous studies have fallen short in two significant ways: accurately recapitulating the phenotype of recurrent patient tumors and identifying an actionable target for increasing therapeutic efficacy.

To better address these issues, we developed a clinically relevant model of TMZ resistance, GBM6R, which was generated in vivo from an orthotopic patient-derived xenograft (PDX) of primary glioblastoma (GBM6) that had undergone multiple rounds of temozolomide (TMZ) and radiation in vivo [9]. Investigation into the molecular differences between these models identified several potential targets, including DNA-PK (*PRKDC*) and the nuclear lamina protein lamin A/C (*LMNA*), involved in the repair of DNA DSBs.

*PRKDC*, which encodes the catalytic subunit of DNA-PK, is a key player in the non-homologous end-joining (NHEJ) pathway for DNA damage response (DDR). It is a pivotal mediator of genome integrity, coordinating repair processes while contributing to transcriptional regulation, telomere maintenance, and apoptosis. *LMNA*, a structural component of the nuclear lamina, supports nuclear integrity and chromatin organization. Like DNA-PK, *LMNA* is involved in DNA repair via the NHEJ pathway [10–13].

This study demonstrates that increased LMNA expression and elevated physical interaction with *PRKDC* in recurrent GBM leads to highly efficient DNA repair mediated by DNA-PK. Based on this finding, chemical inhibition of DDR through DNA-PK inhibition could present a promising therapeutic strategy for treating recurrent GBM.

## Materials and methods

### Cell culture

U251 and HB1 cells were obtained from American Type Culture Collection (Manassas, VA, USA). GBM5, GBM6, GBM6R, and GBM43 were gifted from the James lab. All cells were cultured at 37 °C in 5% CO2 in 1% (for GBM PDX lines) or 10% (for U251 and HB1) fetal bovine serum (FBS; Atlanta Biologicals, Lawrenceville, GA, USA) in Dulbecco’s Modified Eagle’s Medium (DMEM; HyClone, Thermo Fisher Scientific, San Jose, CA, USA) supplemented with 1% penicillin-streptomycin antibiotic mixture (Cellgro, Herndon, VA, USA; Mediatech, Herndon, VA, USA). All GBM cell lines were propagated in the flanks of athymic nude mice and only cultured under in vitro conditions for experimental purposes. Our team has developed and published models of therapy-resistant recurrent glioblastoma [9]. Mechanistic dissection centered on the isogenic GBM6/GBM6R pair to model therapy-adapted recurrence while minimizing inter-tumoral confounders; GBM5 (mesenchymal-leaning) and GBM43 (proneural-leaning) served as pre-specified generalizability checks across distinct molecular states.

### Cell viability

Cell viability assays were performed in biological triplicates, with at least one independent replication for each experiment. Cells were trypsinized following treatment completion (for drug susceptibility assays) or on sequential days (for proliferation assays) and counted using a 1:1 dilution of cell suspension with 0.05% trypan blue solution.

### Animal studies

Athymic nude mice were obtained from Charles River Laboratories and housed in humidity-controlled 12-h light-dark cycles for the duration of all experiments. Animals were anesthetized with a ketamine/xylazine solution for all surgical procedures and injected intracranially with 150,000 cells in the right hemisphere.

KU57788 was diluted in a 5% Tween-80/45% PEG-400/50% PBS solution to the appropriate concentrations and injected intraperitoneally. TMZ for in vivo use was diluted in 5% DMSO/95% PBS to 2.5 mg/kg and also injected intraperitoneally.

### Flow cytometry

Following completion of treatments, cells were trypsinized, washed several times in PBS, fixed in ethanol at 4 °C for 30 min (or conjugated primary antibody solutions at room temperature), and washed again immediately following fixation. For cell cycle FACS, cells were incubated in 1:50 dilution of propidium iodide just before being run through the BD LSRFortessa 6-laser cytometer for analysis. All cytometric analysis was done using FlowJo software.

### qPCR

Following completion of treatments, cells were trypsinized, and mRNA was extracted using the RNeasy Plus kit from Qiagen. cDNA was generated using the iScript cDNA synthesis kit from BioRad. qPCRs were set up as biological duplicates and technical triplicates in 10 μL reactions and run using CFX Connect Real-Time PCR Detection System from BioRad.

For detailed Materials and methods, please see our supplementary material.

## Results

### In vivo generation of recurrent GBM recapitulates a therapy-resistant phenotype

**Fig. 1:**
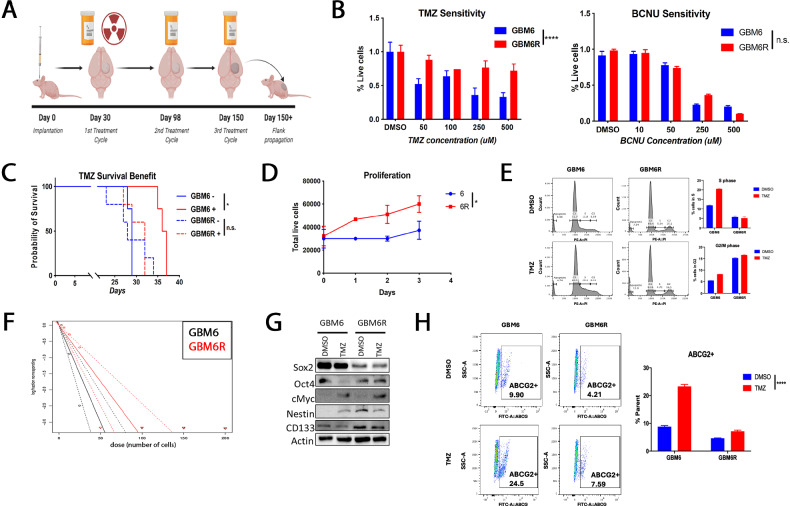
Characterization of the recurrent GBM6R glioblastoma model and its therapeutic response. **A** Schematic of recurrent tumor model generation. GBM6-bearing mice received radiation followed by three cycles of temozolomide (TMZ), resulting in recurrent, treatment-resistant GBM6R tumors. **B** Cell viability assays showing dose-dependent responses of GBM6 (blue) and GBM6R (red) cells to TMZ (left) and BCNU (right). GBM6R cells exhibit TMZ-specific resistance (*p* < 0.0001), while both lines demonstrate similar BCNU sensitivity (*p* = 0.0800). **C** Kaplan–Meier survival curves of mice with intracranial GBM6 or GBM6R tumors treated with TMZ (2.5 mg/kg/day). TMZ significantly prolongs survival in GBM6-bearing mice (*p* = 0.0100), but not in GBM6R-bearing mice (*p* = 0.8325). Blue = vehicle; red = TMZ; solid = GBM6; dashed = GBM6R. **D** Proliferation assay showing increased cell growth over 72 h in GBM6R compared to GBM6 (*p* = 0.0400). **E** Cell cycle analysis of propidium iodide–stained GBM6 and GBM6R cells (>5000 events/condition). TMZ-treated GBM6 cells (50 µM) show S phase accumulation and reduced G2/M populations; GBM6R cells exhibit no significant changes. **F** Extreme limiting dilution assay assessing sphere-forming capacity reveals no significant differences between GBM6 and GBM6R. **G** Western blot analysis of stemness-associated markers (Sox2, Oct4, c-Myc, Nestin, CD133) in GBM6 and GBM6R cells treated with 50 µM TMZ shows no consistent expression differences. **H** Flow cytometric analysis of ABCG2 expression using FITC-conjugated antibody. TMZ increases ABCG2+ populations in both GBM6 (*p* < 0.0001) and GBM6R (*p* = 0.0010) compared to vehicle control.

We have previously published models of therapy-resistant recurrent glioblastoma [9]. Briefly, athymic nude mice were injected intracranially with the parental patient-derived xenograft line GBM6 and, following tumor engraftment (as measured by Bioluminescence Imaging, BLI), treated with one complete cycle of TMZ and concomitant radiation. Upon each recurrence, mice were treated with a TMZ cycle until it became ineffective. Then the tumors were isolated and propagated in the flanks of new mice (Fig. 1A). TMZ sensitivity was assessed in vitro and in vivo (*p* = 0.01), and GBM6R showed more than a two-fold increase in survival over GBM6, but no significant difference in response to another chemotherapy, Carmustine (Fig. 1B). Despite acquired TMZ resistance, GBM6R remains sensitive to carmustine, indicating a lack of cross-resistance between alkylators. Moreover, TMZ significantly prolonged the survival of GBM6-bearing mice, but not GBM6R (Fig. 1C). Together, these data confirm that GBM6R is TMZ resistant.

Proliferation rate analysis showed GBM6R as significantly more proliferative than GBM6 (*p* = 0.04; Fig. 1D). Cell cycle FACS analysis displayed a significantly higher proportion of GBM6 cells in the S phase than G2/M compared to GBM6R (Fig. 1E). Next, expression of critical oncogenic and resistance drivers in GBM, such as p53, MGMT, c-MYC, cancer stem cell markers CD133, Sox2, and metabolic regulator IMPDH2 was examined over time in response to TMZ with no significant difference except downregulated IMPDH2 and β-catenin in GBM6R (Supplementary Fig. 1A). We observed no difference in sphere-forming capacity between GBM6 and GBM6R, nor a consistent increase in canonical stem-like markers (Fig. 1F, G). Finally, we explored the MDR transporter ABCG2 in our resistance model due to its well-established role in therapy resistance [11, 12]. We found a significant increase in the percentage of cells expressing ABCG2 following TMZ in GBM6 (p < 0.0001) and GBM6R (*p* = 0.001) (Fig. 1H).

### Proteomic characterization of recurrent GBM demonstrates several potential targets of therapeutic resistance

**Fig. 2:**
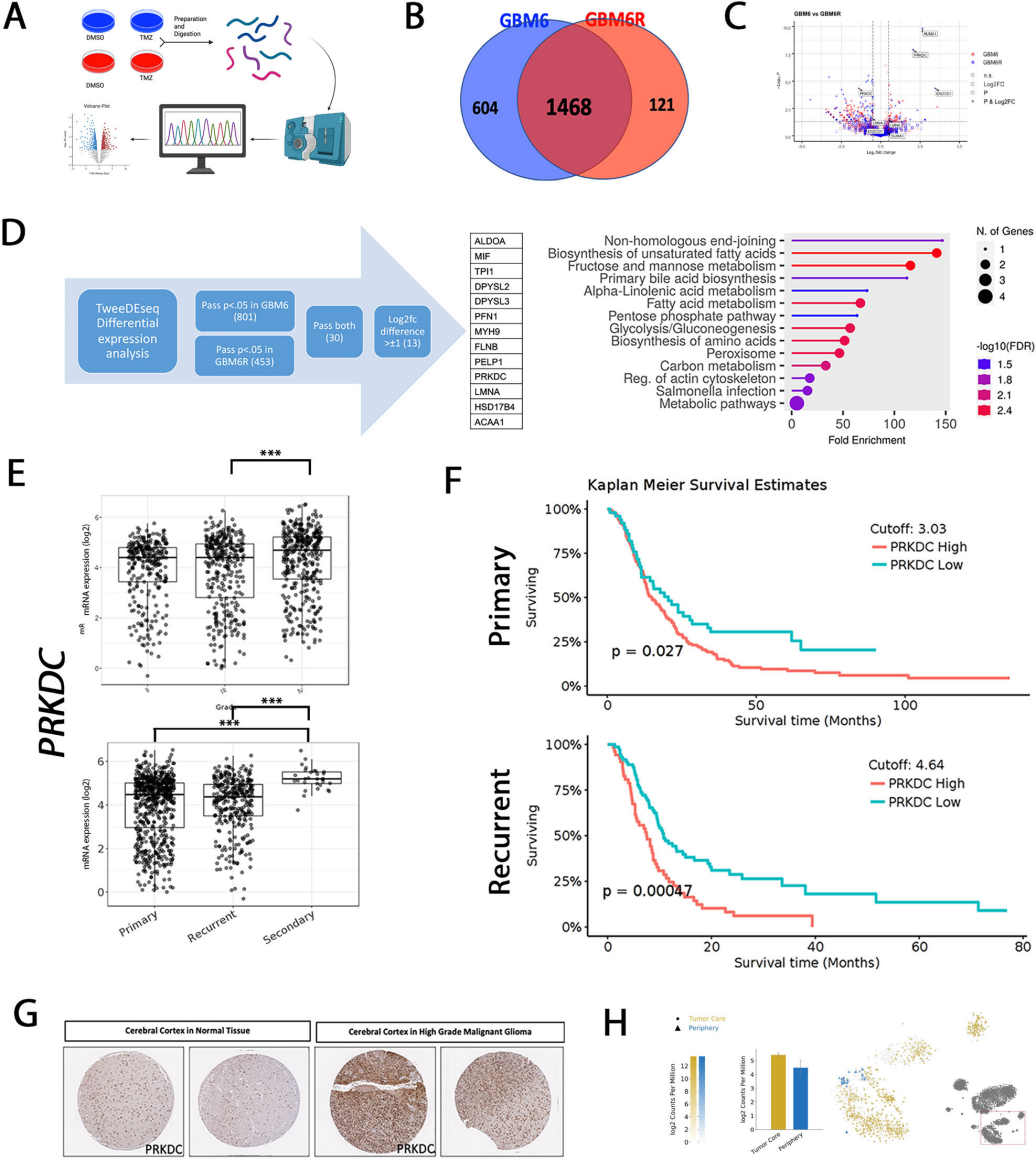
Unbiased proteomic profiling reveals TMZ-associated upregulation of LMNA and PRKDC in recurrent GBM and their correlation with patient datasets. **A** Schematic of the experimental workflow. GBM6 and GBM6R cells were treated with temozolomide (TMZ, 50 µM) or DMSO control and subjected to bottom-up proteomic analysis using liquid chromatography–tandem mass spectrometry (LC-MS/MS). **B** Venn diagram of differentially expressed proteins across treatment groups. Of the ~1500 proteins identified, 121 were significantly upregulated in GBM6R relative to GBM6. **C** Volcano plot generated with tweeDEseq package shows differentially expressed proteins between GBM6 and GBM6R. Proteins with *p* < 0.05 and |Log₂ fold change | > 0.5 were considered significant and prioritized for downstream analysis. **D** Selection of the top 13 differentially expressed genes based on tweeDEseq analysis, with KEGG pathway enrichment visualized in a dot plot. **E** Analysis of CGGA datasets via GlioVis. PRKDC mRNA expression is elevated in high-grade gliomas and secondary GBM compared to low-grade gliomas, with statistical significance assessed by pairwise *t*-test (p < 0.001). **F** Kaplan–Meier survival curves from CGGA cohorts. Both primary (*p* = 0.027) and recurrent (*p* = 0.00047) GBM samples show improved survival in patients with low PRKDC expression. **G** Immunohistochemistry from the Human Protein Atlas show elevated *PRKDC* protein levels in malignant cortical tissue relative to normal cortex. **H** Single-cell transcriptomic data from the GBMseq dataset localize *PRKDC* expression to malignant cells in both the tumor core and infiltrative periphery.

To investigate the mechanism of chemoresistance, we conducted a large-scale, unbiased proteomics analysis to capture protein expression between TMZ-treated sensitive GBM6 and resistant GBM6R using liquid chromatography-tandem mass spectrometry (LC-MS/MS; Fig. 2A). After removing duplicate GeneIDs from this data, roughly 1500 proteins were identified in both GBM6 and GBM6R treatment conditions (Fig. 2B). Using significance cutoffs of *p* < 0.05 and Log 2-fold change cutoffs of FC > ± 0.5, 13 proteins were significantly upregulated in the therapy-resistant GBM6R in response to TMZ (Fig. 2C). Next, using a combination of Cytoscape and ShinyGO 0.80, Gene Set Enrichment Analysis identified the “Metabolic Pathway” (FDR = 0.008), specifically the “Fatty Acid” metabolism (FDR = 0.006) and the “Non-homologous end-joining” (FDR = 0.02) as enriched in the resistant GBM6R in response to TMZ compared to the parent PDX line, GBM6 (Fig. 2D).

Of these 13 genes, *PRKDC*, a gene encoding the catalytic subunit of DNA-dependent protein kinase, showed elevated expression in the grade IV patients’ cohort and patients with low *PRKDC* expression had significantly better survival than those with high expression, not seen in the other genes (Fig. 2E, F, Supplementary Fig. 1B; http://gliovis.bioinfo.cnio.es/). qPCR validation confirmed *PRKDC’s* elevated transcript levels in GBM6R compared to GBM6 in a TMZ-dependent manner (Supplementary Fig. 2A). Protein level *PRKDC* expression was also significantly elevated in GBM tissues compared to normal brain tissue (Fig. 2G). Analysis of publicly available GBM patient datasets from GBMseq showed cells with elevated PRKDC expression were concentrated in malignant cells within the tumor core and periphery (Fig. 2H). In GBmap, *PRKDC* is enriched in malignant cells with low–modest expression across microglia/macrophages, oligodendrocytes, astrocytes, endothelial, and perivascular lineages (Supplementary Fig. 3A).

### *PRKDC* inhibition sensitizes the resistant PDX line to additional TMZ therapy

**Fig. 3:**
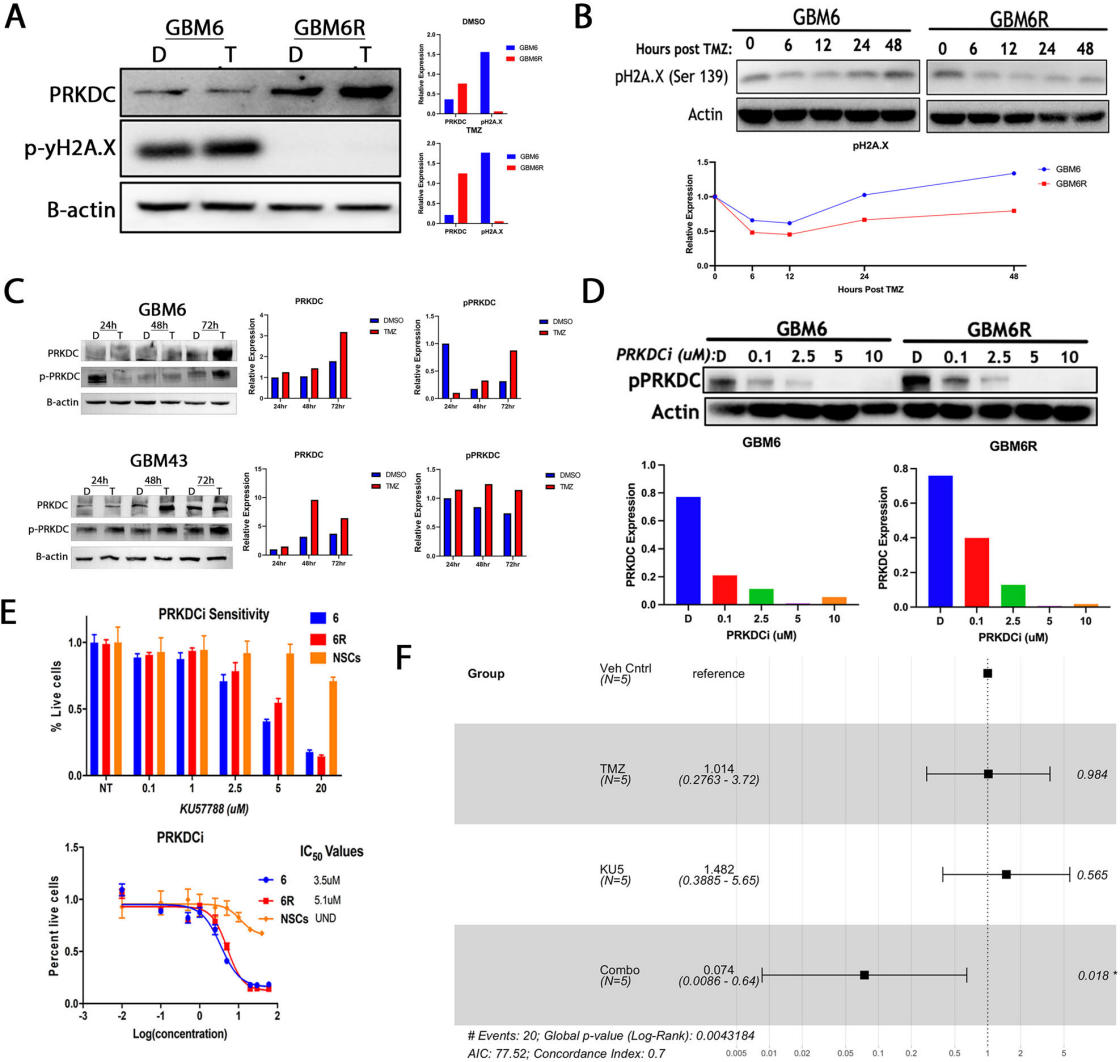
*PRKDC* expression and pharmacologic inhibition in GBM6/GBM6R models: effects on DNA damage response and therapeutic sensitivity. **A** Immunoblot analysis of GBM6 and GBM6R cells treated with DMSO or TMZ (50 µM). GBM6R cells show elevated *PRKDC* expression and reduced levels of phosphorylated γH2A.X (pH2A.X), a DNA damage marker, compared to GBM6 in both conditions. **B** Time-course immunoblot of TMZ-treated cells (0–48 h). GBM6 and GBM6R cells show progressive increases in total *PRKDC* and phosphorylated *PRKDC* (p-PRKDC) expression across multiple timepoints. **C** Immunoblotting of GBM6 and GBM43 cells at 24, 48, and 72 h post-TMZ exposure shows dynamic regulation of *PRKDC* and p-PRKDC in response to DNA damage. **D** Dose-dependent effects of *PRKDC* inhibitor KU57788 (0.1–10 µM) reduces p-PRKDC levels in both GBM6 and GBM6R cells. **E** Cell viability assays reveal that GBM6 and GBM6R cells are more sensitive to KU57788 (IC₅₀ ~5 µM) than non-malignant neural stem cells (NSCs), indicating selective tumor cytotoxicity at escalating inhibitor concentrations. **F** Survival analysis of mice orthotopically implanted with GBM6R cells and treated with DMSO vehicle, TMZ (2.5 mg/kg), KU57788 (10 mg/kg), or a combination of TMZ and KU57788. Forest plot demonstrates that combination treatment significantly prolongs survival compared to all other groups (hazard ratio: 0.074; 95% confidence interval: 0.0086–0.64; *p* = 0.018).

*PRKDC*, the catalytic subunit of DNA-dependent protein kinases (DNA-PK), works with Ku70/Ku80 heterodimer protein to repair DNA double-strand breaks by phosphorylating H2AX at Ser139 (γH2AX) and aids complex formation [14]. *PRKDC* expression is consistently elevated in the TMZ-resistant GBM6R line, which increases following TMZ exposure (Fig. 3A). GBM6 displayed a sustained DDR with persistent γH2AX (Ser139) and upstream activation (p-ATM/ATR, p-Chk1/2, p-p53) over 24–72 h, whereas GBM6R showed a transient γH2AX spike that diminished by 48–72 h under identical TMZ conditions—consistent with attenuated maintenance of canonical DSB signaling in GBM6R (Fig. 3A, B). Concordantly, a DDR phospho-panel showed TMZ-induced phosphorylation of ATM (Ser1981), ATR (Ser428), BRCA1 (Ser1524), CHK1 (Ser345), CHK2 (Thr68), p53 (Ser15), and γH2AX (Ser139) in GBM6, with attenuated maintenance in GBM6R (Supplementary Fig. 3B). Thus, LMNA levels modulate the rate of γH2AX resolution, linking LMNA–PRKDC engagement to enhanced repair capacity under TMZ. In other PDX models, the dynamic of *PRKDC* phosphorylation post-TMZ exposure varied significantly, indicating this disease’s heterogeneous nature (Fig. 3C). In GBM6, both total *PRKDC* and phospho-*PRKDC* reached the maximum at 72 h post TMZ exposure, but for GBM43, both reached the maximum at 48 h.

To investigate *PRKDC*’s response to TMZ therapy, we employed KU57788, an ATP-competitive inhibitor of DNA-PK with a Ki value of 0.65 nM. KU57788 effectively blocks the phosphorylation-dependent activation of PRKDC in a dose-dependent manner with complete inhibition at 5 µM (Fig. 3D). KU57788 kills both GBM6 and, most importantly, the therapy-resistant GBM6R cell line with IC₅₀ values of 3.5 μM and 5.1 μM, respectively (Fig. 3E). Neural stem cells treated with KU57788 observed significantly less killing, indicating *PRKDC* and DNA-PK are crucial for mounting a DNA damage response in GBM. Although several DNA-PK inhibitors (e.g., peposertib, AZD7648) are in clinical testing, our head-to-head assays showed that KU57788 produced greater on-target cytotoxicity than AZD7648 under matched conditions (AZD7648 did not reach an IC₅₀ within 0–20 µM; Supplementary Fig. 3C, D). We therefore used KU57788 as a potent tool compound to define DNA-PK dependence while noting that clinical translation will require human-tested agents.

Finally, we investigated the combination of KU57788 with TMZ in vivo by orthotopically implanting GBM6R cells and waiting for tumor establishment. After 10 days, animals were treated for 5 days with KU57788, TMZ, or combined and monitored for end-point survival. We found that the combination treatment significantly reduced the hazard (HR = 0.074, 95% CI: 0.0086–0.64, *p* = 0.018), lowering the risk by 92.6% compared to the vehicle control group (Fig. 3F).

### *PRKDC* interacts with *LMNA* in response to TMZ in chemoresistant GBM cells

**Fig. 4:**
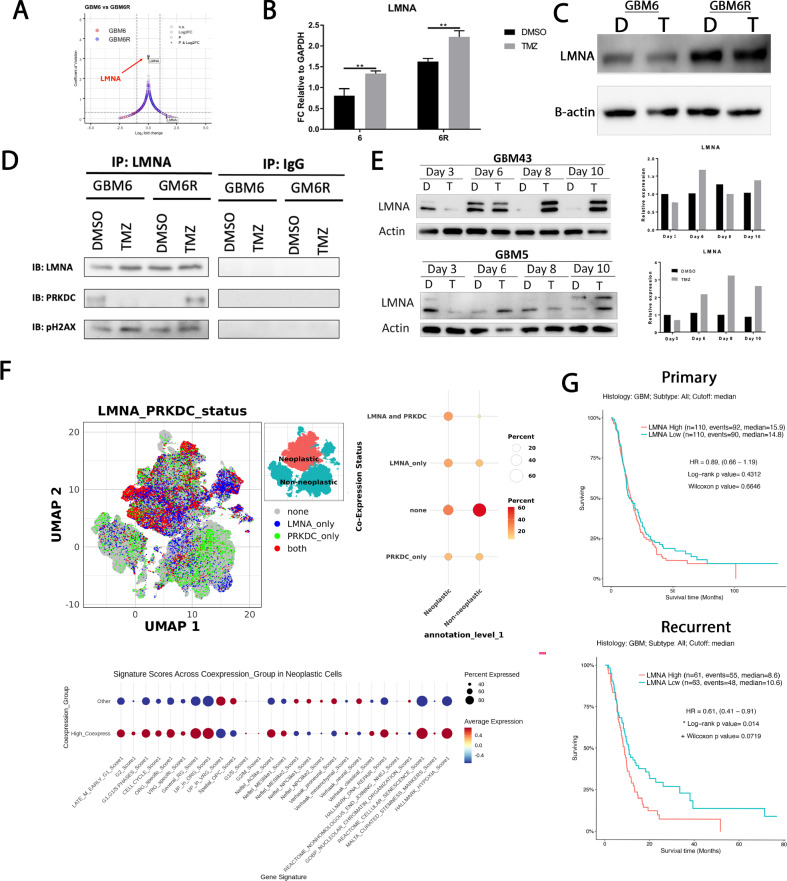
*LMNA* expression is induced by TMZ and co-expressed with *PRKDC* in malignant glioblastoma cells, correlating with poor patient survival. **A** Volcano plot from proteomic profiling. GBM6 and GBM6R cells treated with temozolomide (TMZ, 50 µM) or DMSO control were subjected to bottom-up proteomics via liquid chromatography–tandem mass spectrometry (LC-MS/MS), highlighting LMNA as significantly upregulated. **B** RNA sequencing analysis shows a significant increase in LMNA transcript levels following TMZ treatment in both GBM6 and GBM6R cells. **C** Immunoblot analysis confirms upregulation of *LMNA* protein in response to TMZ in both GBM6 and GBM6R cells. **D** Immunoprecipitation shows TMZ-induced LMNA–PRKDC complex formation. This assay reports interaction, not global DNA damage; lower input γH2AX in GBM6R reflects faster repair, not absence of engagement. **E** Time-course immunoblot analysis of patient-derived GBM43 and GBM5 cells reveals progressively increased *LMNA* expression following repeated TMZ treatments. **F** Single-cell transcriptomic analysis of GBmap dataset. (Top) UMAP clustering of *LMNA*⁺ (blue), *PRKDC*⁺ (green), and *LMNA*⁺/*PRKDC*⁺ (red) cells in non-malignant and malignant populations from the GBmap single-cell transcriptomic dataset. (Bottom) Dot plot summarizing the distribution of *LMNA*-only, *PRKDC*-only, double-positive, or double-negative cells across distinct malignant GBM cell types. **G** Kaplan–Meier survival analysis from the CGGA dataset, accessed via GlioVis, indicates significantly improved survival in patients with low *LMNA* expression in both primary and recurrent GBM cohorts.

Further investigation and IP-MS analysis identified *LMNA* as a significant binding partner of *PRKDC* during TMZ therapy in the chemoresistant GBM6R cell line (Fig. 4A). We then explored a potential link between *LMNA* and DNA damage repair in recurrent GBM, given the unknown role of *LMNA* in TMZ resistance. *LMNA* mRNA expression was significantly elevated during TMZ therapy in both TMZ-sensitive and resistant lines (Fig. 4B). The TMZ-resistant GBM6R line has significantly elevated *LMNA* protein expression compared to GBM6 regardless of TMZ exposure (Fig. 4C). To validate our IP-MS analysis, we performed immunoprecipitation analysis and observed that the interaction between *LMNA* and *PRKDC* was significantly reduced during TMZ therapy in GBM6, while significantly increased in GBM6R (Fig. 4D). *LMNA*-*PRKDC* interaction was only present during TMZ therapy. *LMNA* expression in another molecular subtype of PDX models was absent early post-TMZ exposure (Fig. 4E). However, *LMNA* expression (top band lamin A, bottom band lamin C) was elevated after day 8 for GBM43 (proneural) and day 10 for GBM5 (mesenchymal) with 2 and 4 repeated TMZ exposures (50 μM). GBM6/6R exhibit higher baseline LMNA with modest modulation, whereas GBM5/GBM43 show delayed, exposure-accumulated induction—reflecting subtype- and kinetics-dependent regulation. In GBM6R, accelerated repair yields lower steady-state γH2AX in inputs despite increased LMNA–PRKDC interaction, consistent with DDR kinetics in Fig. 3A, B and loss of upstream DDR phosphorylation over time (Supplementary Fig. 3B).

Analysis of GBmap, a publicly available single-cell RNA sequencing dataset of 338,564 cells from 110 GBM patients [15], revealed that coexpression is highest in malignant cells, specifically oligodendrocyte and neural progenitor cell-like population (Fig. 4F). *LMNA* expression was negatively correlated with survival in recurrent GBM, according to the GlioVis Chinese Glioma Genome Atlas dataset (Fig. 4G).

### In vivo characterization of GBM cells co-expressing *LMNA* and *PRKDC* during TMZ therapy

**Fig. 5:**
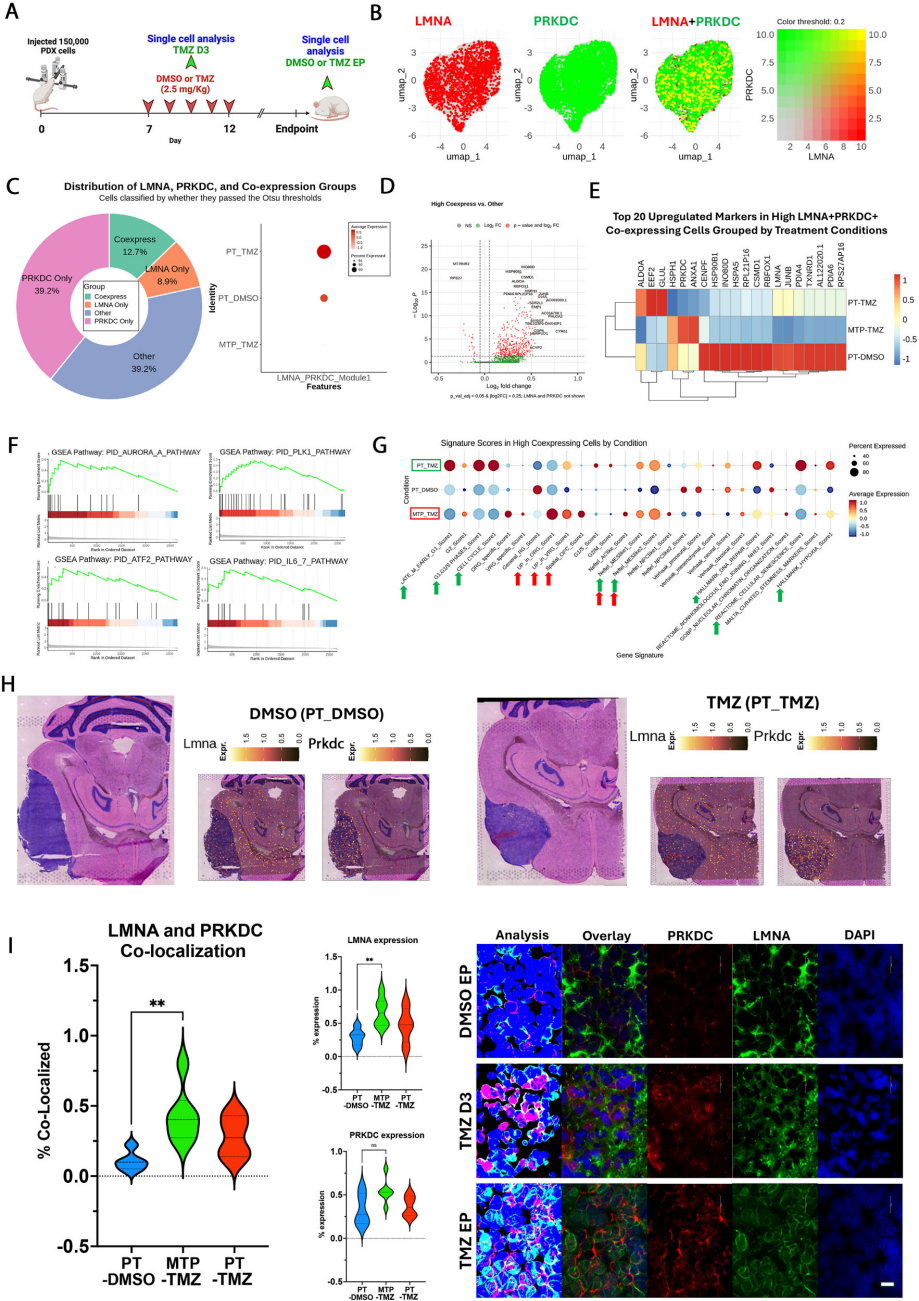
Co-expression of *LMNA* and *PRKDC* defines a distinct GBM subpopulation with unique transcriptional and epigenetic characteristics. **A** Schematic of the in vivo experimental design. Mice bearing intracranial GBM tumors were treated with DMSO or temozolomide (TMZ, 2.5 mg/kg). Brain tissues were harvested on Day 3 of treatment or at survival endpoint for single-cell RNA sequencing (scRNA-seq). **B** UMAP visualization of integrated scRNA-seq highlighting *LMNA* + (red), PRKDC+ (green), and LMNA + /PRKDC+ (yellow) co-expressing tumor cell populations. **C** Donut chart quantifying the proportion of LMNA-only, PRKDC-only, LMNA-PRKDC double-positive (Coexpress), and other cells versus all other cells in the dataset. (“Expressed” = non-zero UMI; “co-high” = gene-wise *z* ≥ 1.0 for both *LMNA* and *PRKDC* using a single fixed gate across treatment groups. Cells from PT_DMSO, PT_TMZ, and MTP_TMZ were integrated with batch correction; quantifications in 5 C are per-group proportions computed on the integrated object). **D** Volcano plot depicting differentially expressed genes between the LMNA-PRKDC co-expressing (“High Coexpress”) cluster and all other tumor cells. The x-axis shows log₂ fold change, and the y-axis shows –log₁₀ *p*-values (statistical test: Wilcoxon rank-sum). **E** Heatmap showing the top 20 marker genes enriched in the High Coexpress subpopulation stratified by treatment groups. **F** Gene set enrichment analysis (GSEA) identifies the top four pathways significantly enriched in the High Coexpress population. **G** Dot plot illustrating module scores across cell populations stratified by treatment, revealing unique transcriptional programs enriched in High Coexpress cells. **H** Spatial feature plots demonstrating regional expression patterns of *LMNA* and *PRKDC* in mouse brain sections after DMSO (left) or TMZ (right) treatment. **I** Violin plots show increased *LMNA* expression and *LMNA-PRKDC* overlap in TMZ-treated (MTP_TMZ, PT_TMZ) versus DMSO-treated (PT_DMSO) brains. Immunofluorescence demonstrates co-localization of *PRKDC* (red) and *LMNA* (green) in tumor.

To characterize *LMNA*–*PRKDC* co-expressing cells in vivo, we profiled the GBM43 PDX by single-cell RNA sequencing (scRNA-seq). After 7 days of implantation, mice received 5 days of vehicle (PT_DMSO) or TMZ (PT_TMZ) and were harvested at disease endpoint; an additional cohort was collected mid-therapy after 2 days of TMZ (MTP_TMZ) (Fig. 5A). Single-cell suspensions from tumor-bearing brains were processed by Drop-seq and analyzed with a custom Seurat v3 workflow to quantify *LMNA*, *PRKDC*, and their co-expression (Fig. 5B; Supplementary Fig. 4A). Overall, 12.3% of malignant cells were co-high for *LMNA* and *PRKDC*, peaking in post-therapy tumors (PT_TMZ) (Fig. 5C). The top co-high markers were *INO80D* (chromatin remodeling), *HSP90B1* (chaperone), and *CSMD1* (complement regulation) (Fig. 5D). These genes were most enriched in vehicle-treated controls (PT_DMSO), reflecting vehicle-state chromatin/chaperone programs (Fig. 5E), whereas TMZ arms shifted toward cell-cycle/DDR signatures, consistent with γH2AX kinetics. Among the top co-high markers (INO80D, HSP90B1, CSMD1), expression was most enriched under PT_DMSO. These chromatin remodeling and chaperone programs likely represent a baseline state that pre-primes the co-high subpopulation for rapid DDR and cycling upon TMZ exposure, as seen with GSEA trends in Fig. 5F. GSEA of the top 20 co-high markers showed enrichment for Aurora-A, PLK1, ATF2, and IL-6 signaling (Fig. 5F; Supplementary Fig. 4B, C) [16]. Comparison with MSigDB indicated that co-high cells align with hypoxia-dependent mesenchymal-like GBM (Neftel_MESlike2) in PT_TMZ and MTP_TMZ; during therapy, they also exhibit outer radial glia and OPC-like features alongside active cell-cycle and DNA-repair programs (Fig. 5G; Supplementary Fig. 4D). Spatial transcriptomics confirmed *LMNA* and *PRKDC* expression in the GBM43 PDX (Fig. 5H). *LMNA*–*PRKDC* co-expression increased after therapy and was validated by IHC co-staining, with colocalization significantly elevated during TMZ treatment (Fig. 5I; Supplementary Fig. 4E).

### *LMNA* expression confers resistance to TMZ by enhancing DNA repair capacity in GBM

**Fig. 6:**
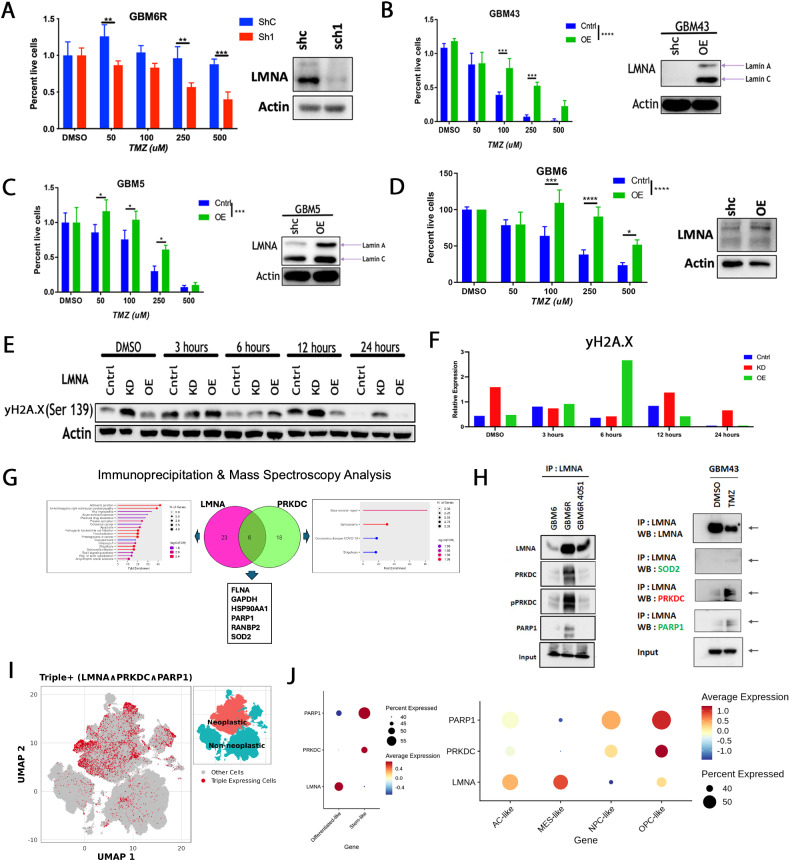
Lamin A/C expression mediates temozolomide (TMZ) resistance in glioblastoma models. **A** *LMNA* knockdown (KD) restores TMZ sensitivity in GBM6R cells. Cell viability is significantly reduced in *LMNA* KD (Sh1) cells compared to scrambled control (ShC) cells following TMZ treatment (*p* < 0.0001). Immunoblot confirms effective *LMNA* knockdown via lentiviral transduction. *LMNA* overexpression (OE) induces TMZ resistance in patient-derived primary GBM43 (**B**), GBM5 (**C**), and GBM6 (**D**) cells. Bar graphs show increased cell viability in *LMNA* OE cells compared to controls after TMZ treatment. Right panels: Immunoblots confirm successful *LMNA* overexpression in each line. **E** Immunoblot analysis of phosphorylated γH2A.X (γH2A.X) levels following TMZ. *LMNA* KD and control cells sustain DNA damage, whereas *LMNA* OE cells exhibit reduced γH2A.X, indicating enhanced DNA repair capacity. **F** Schematic model depicting *LMNA*-mediated TMZ resistance via interaction with DNA damage response proteins, including *PRKDC* and γH2A.X, during GBM recurrence. **G** Mass spectrometry of *LMNA* immunoprecipitates from TMZ-resistant GBM6R cells identifies six proteins that overlap as interaction partners for both *LMNA* and *PRKDC*. **H** Left: Immunoblot of *LMNA* immunoprecipitates from GBM6, GBM6R, and GBM6R 4051 samples reveals co-precipitation of *PRKDC*, phosphorylated *PRKDC* (p-PRKDC), *PARP1*, and *LMNA*. Input controls are shown. Right: Immunoblot of *LMNA* immunoprecipitates from GBM43 cells treated with DMSO or TMZ shows interaction with *SOD2, PRKDC, PARP1*, and *LMNA*. **I** UMAP plot of scRNA-seq data highlights expression of *LMNA*, *PRKDC*, and *PARP1* within the neoplastic cell compartment. **J** Left: Quantification of *LMNA*, *PRKDC*, and *PARP1* expression across differentiated-like versus stem-like glioblastoma cells. Right: Expression stratified by Neftel transcriptional states shows enrichment of these genes within transcriptional programs associated with therapy resistance.

*LMNA* encodes lamin A, a protein essential for nuclear structure and gene regulation, but its role in GBM development and progression remains unknown. To examine if *LMNA* confers resistance to TMZ in our PDX model, we knocked down (KD) *LMNA* in the TMZ resistant GBM6R line using shRNA and observed that *LMNA* KD sensitized cells to TMZ (Fig. 6A). In contrast, when we overexpressed *LMNA* in different subtypes of PDX, all PDX lines acquired resistance to TMZ (Fig. 6B–D). When we KD or OE *LMNA* in immortalized normal neural stem cell HB1.F3.CD line, we observed no change in the growth (Supplementary Fig. 5A) [17]. As *LMNA* interacts with *PRKDC* in the resistance line, and *PRKDC* is the catalytic subunit of DNA-PK, a crucial component of the DNA repair pathway, we next investigated if LMNA contributes to DNA repair post-TMZ therapy by measuring the γH2AX in our TMZ-resistant GBM6R line (Fig. 6E and Supplementary Fig. 5B). The data indicated that the GBM cells’ ability to resolve TMZ-induced γH2AX foci was significantly impaired in the *LMNA* KD cells compared to the control. Conversely, *LMNA* overexpressing cells can rapidly resolve the γH2AX foci post TMZ therapy (Fig. 6F).

We next examined our IP-MS results to further investigate the possible mechanism by which *LMNA* and *PRKDC* promote resistance to TMZ (Fig. 6G). Six proteins were identified as interacting with both *LMNA* and *PRKDC* in our TMZ-resistant GBM6R line. To validate these targets, we performed immunoprecipitation with LMNA antibody followed by immunoblot analysis of the target proteins. We found only PARP1 as a possible interactor with *LMNA* and *PRKDC* in our resistance line (Fig. 6H). Next, to examine if such interaction can occur during TMZ therapy, we treated the GBM43 PDX line with DMSO or TMZ and performed immunoprecipitation analysis. Our data indicated that during TMZ therapy, *LMNA* can interact with *PRKDC* and PARP1 (Fig. 6H, left).

In clinical samples, single-cell RNA sequencing analysis revealed that the malignant cells could express all three transcripts simultaneously (Fig. 6I and Supplementary Figs. 5C, 4D). These cells expressing *LMNA*, *PRKDC*, and *PARP1* transcripts are molecularly more dedifferentiated glioma stem-like cells, specifically OPC-like cells (Fig. 6J). Based on this, we concluded that the *LMNA*-*PRKDC* axis can contribute to therapeutic resistance in GBM.

## Discussion

Therapeutic resistance is a hallmark of recurrent GBM, yet there is no effective treatment, largely due to a lack of proven resistance mechanisms consistent across GBM subtypes. Utilizing a clinically relevant resistance model of GBM, we identified the role of the *LMNA*-*PRKDC* axis in TMZ resistance. Characterizing the genetic contributions underlying chemotherapeutic resistance is key to more robust preclinical data resulting in better patient care. Most of the recurrent GBM tumor cell biology has been studied at the patient-tissue level. While MGMT promoter methylation status and some driver mutations predict therapeutic success to some degree, they do not apply to all recurrent GBMs [6, 18]. The standard of care for recurrent GBM patients includes secondary surgical resection of the tumor, a procedure for which only 50% of patients are eligible. Chemotherapeutic rechallenging, re-irradiation, and tumor-treating fields have, at best, only modest effects.

To investigate chemoresistance mechanisms and recurrent GBM biology, we generated an IDH-wildtype recurrent PDX (GBM6R) via repeated in vivo standard chemoradiation (RT + TMZ) [9]. GBM6R’s preserved sensitivity to carmustine (BCNU) indicates that TMZ tolerance is pathway-specific rather than global chemoresistance. TMZ lethality is driven by replication-dependent O⁶-methylguanine/mismatch-repair signaling, whereas BCNU kills through interstrand cross-links resolved by MGMT and Fanconi anemia/homologous recombination (FA/HR) pathways [16, 17, 19, 20]. Consistent with this, we infer that LMNA–PRKDC–driven tolerance is selective for TMZ-type lesions. Although ABCG2 is higher in GBM6R, available data suggest it is unlikely to explain the phenotype: in GBM cells ABCB1 dominates intracellular TMZ efflux, while ABCB1/ABCG2 chiefly restrict brain entry at the BBB—an exposure effect that cannot account for within-tumor GBM6 vs GBM6R differences [21, 22]. Together with *LMNA*–*PRKDC* engagement and preserved BCNU sensitivity, these findings support lesion-pathway–specific TMZ tolerance rather than transporter-mediated resistance; nonetheless, dual P-gp/BCRP inhibition or *ABCB1*/*ABCG2* knockdown would definitively exclude transporter contributions. Using this model, we next applied unbiased LC-MS/MS and functional assays, revealing a previously unrecognized *LMNA*–*PRKDC* interaction that drives TMZ resistance in recurrent GBM. Across independent PDX backgrounds, we observed directional concordance for four sentinel readouts: (i) TMZ → *PRKDC* activation (GBM6, GBM43; Fig. 3C); (ii) repeated TMZ → *LMNA* induction (GBM5, GBM43; Fig. 4E); (iii) LMNA gain-of-function→TMZ tolerance (GBM6, GBM5, GBM43; Fig. 6B–D); and (iv) TMZ → *LMNA*–*PRKDC* complex formation (GBM43; Fig. 6H). Despite expected kinetic heterogeneity across models, these cross-line data support a mechanism that is not restricted to a single GBM subtype.

Mammalian double-strand breaks are repaired predominantly by canonical non-homologous end joining (c-NHEJ), coordinated by the Ku–DNA-PKcs (PRKDC) complex. Upon DSB detection, DNA-PKcs kinase activity—redundant with ATM—phosphorylates H2AX on Ser139 and promotes end-processing/ligation. DNA-PKcs is frequently overexpressed/activated in human cancers, including GBM, and sustains the glioma stem-cell state; its inhibition drives differentiation and sensitizes tumors to therapy [23–29]. In this study, we reported that *PRKDC* is crucial for TMZ resistance in a clinically relevant recurrent PDX model of GBM. The elevated expression of *PRKDC* mRNA is associated with poor outcomes in patients with recurrent GBM. Our data show that the resistance line has an elevated level of *PRKDC* at the resting state, and, in response to TMZ, expression was further elevated. DDR is measured by γH2AX, which was significantly lower in the resistance line. When we blocked the *PRKDC* activity with the small molecule inhibitor, the resistant line became sensitive to TMZ both in vitro and in vivo, indicating the role of *PRKDC* in promoting resistance to TMZ in GBM.

To further investigate the mechanisms of *PRKDC*-mediated chemoresistance, we next performed an IP-mass spectroscopy analysis to identify a unique binding partner of *PRKDC*. This analysis identified lamin A/C, encoded by the *LMNA* gene, as a binding partner for *PRKDC* in our resistance line during TMZ therapy. *LMNA*, the genetic encoder for lamin A/C proteins, confers nuclear membrane stability, cytosolic stability, cell motility, and DDR capacity, ultimately providing cells with the necessary structural frameworks for survival [30, 31]. Mutations in the *LMNA* gene result in degenerative disorders, including progeria. In addition, many cancers contain various modifications in *LMNA* contributing to disease progression [32]. Even though *LMNA* is associated with increased aggressiveness in GBM, the precise mechanism has yet to be established [33]. Our data indicated that *LMNA* expression increased after TMZ treatment and knocking down *LMNA* in TMZ-resistant GBM6R sensitized the cells to TMZ. When we overexpressed *LMNA* in the three different molecular subtypes of treatment-naïve PDX lines, cells became resistant to TMZ, indicating its role in promoting chemoresistance.

Various point mutations within the *LMNA* gene lead to the expression of a truncated form of the protein, termed progerin, which is responsible for progeria, a genetic disorder that causes children to age rapidly [34, 35]. The structural defects of LMNA impair the cells’ DSB repair capacity [36]. Mutated *LMNA* cannot bind to DDR proteins, including DNA-PK [37]. However, despite the links between *LMNA* and DNA-PK/*PRKDC* and DNA-PK and DDR, no report has investigated their relationship or its role in chemoresistance in GBM or cancer. Our data indicate that lamin A/C preferentially interacts with *PRKDC*, the catalytic subunit of DNA-PK, in response to DNA alkylating agents and initiates robust DDR to promote chemoresistance in GBM. Our IP-MS analysis revealed that *PRKDC* and *LMNA* could interact with poly(ADP-ribose) polymerase 1 (PARP1) in the chemoresistant PDX line and the treatment-naïve PDX line in response to TMZ. PARP1 is an ADP-ribosylating enzyme involved in several crucial cellular processes, including DNA repair, programmed cell death, and genomic stability [38]. It can be a nick DNA sensor and physically recognize DNA lesions to activate DDR pathways, including base excision repair (BER) [29]. In GBM, PARP1 acts as a sensor that activates the base excision pathway, and inhibition enhances the efficacy of TMZ therapy [39]. Taken together, the interaction between *LMNA* and *PRKDC* in the resistance line and post-TMZ exposure to our treatment-naïve PDX line may contribute to more efficient BER/single-strand break repair and thus contribute to resistance.

Mechanistically, we propose that LMNA scaffolds PRKDC (DNA-PKcs) and PARP1 at DNA lesions to accelerate DSB/SSB repair under alkylation stress. This model predicts that PARP1 inhibition (± DNA-PK inhibition) would abrogate LMNA-mediated tolerance.

GBM cells are highly plastic in their transcriptional signature, allowing them to adapt to various environmental stresses, including therapy [40, 41]. The single-cell RNA sequencing analysis in our in vivo GBM model, as well as clinical samples from GBM patients shows that the *LMNA* and *PRKDC* co-expressing cells exhibited more radial glia and oligodendrocyte precursor cell-like transcriptomic signatures during therapy, which are considered to be plastic. Previous reports show that *PRKDC* is preferentially expressed in glioma stem cell-like populations and stabilizes Sox2, a core transcription factor that maintains the glioma stem cell niche in GBM [21]. Our data align with this finding, indicating that *PRKDC* and *LMNA* expression in the glioma stem cell compartment contribute to TMZ resistance in GBM.

Our study has established a new *PRKDC*-*LMNA* axis that contributes to chemoresistance in GBM by augmenting DDR. Targeting such a signaling cascade with small-molecule inhibitors against DNA-PK reverses the TMZ resistance in clinically relevant recurrent PDX models, thus opening the possibility of clinical translation to overcome chemoresistance in GBM.

## Supplementary information

Supplementary Material

## Data Availability

All data supporting the findings are available within the article and/or its Supplementary Materials. Public datasets used include TCGA (https://www.cancer.gov/tcga), Human Protein Atlas, and GBMSeq. Single-cell sequencing data generated in this study have been deposited in GEO under accession GSE227348.

## Code availability

Custom code used for the bioinformatic analyses is available from the corresponding author upon reasonable request.
