# Supplementary material for: LMNA-PRKDC axis enhances DNA repair and promotes chemoresistance in glioblastoma
Source: Cell Death Dis. 2025 Nov 21;17(1):50. doi: 10.1038/s41419-025-08226-3 (PMC12811359; doi:10.1038/s41419-025-08226-3)
Supplement: Supplementary file 1 — Supplementary Material [file 41419_2025_8226_MOESM1_ESM.docx]

**LMNA-PRKDC axis enhances DNA repair and promotes chemoresistance in glioblastoma**

**Supplementary Material**

Miranda R. Saathoff^⊥^, Rafal Chojak^⊥^, Rebecca X Chen, Hasaan A. Kazi, Umme H Faisal, Jack M Shireman, Noah Drewes, Cheol H. Park, Xuesong Fan, Sana A Khan, Irene Lazanyi, Shivani Baisiwala, James C. David, Craig M. Horbinski, and Atique U. Ahmed*

Department of Neurological Surgery, Feinberg School of Medicine, Northwestern University, Chicago, IL, USA 60616

*To whom correspondence should be addressed:

Atique U Ahmed

Department of Neurological Surgery

Room 3-112

303 E Superior Street

Chicago, IL 60616

Email: [atique.ahmed@northwestern.edu](mailto:atique.ahmed@northwestern.edu)

Phone: +1 (312) 503-3552

^⊥^ These authors contributed equally

**Running title:** LMNA–PRKDC Axis Drives DNA Repair and Chemoresistance in GBM

**Disclosure of Potential conflicts of Interest**

The authors declare no competing financial interests

**Animal Studies**

Athymic nude mice (Foxn1nu strain), male and female, aged 6–8 weeks, were obtained from Charles River Laboratories and maintained in a controlled environment with 12-hour light-dark cycles, 22±2°C temperature, and 50±10% humidity. For surgical procedures, mice were anesthetized using a ketamine/xylazine solution and intracranially injected with 150,000 cells into the right hemisphere. Treatments commenced on post-surgical day 6 and continued for 6 days. A researcher assigned animals to treatment groups (TMZ, KU57788, combination, or vehicle) using a manual randomization process to ensure unbiased allocation. KU57788 was prepared in a 5% Tween-80/45% PEG-400/50% PBS solution and administered intraperitoneally at the specified concentrations. TMZ was diluted in 5% DMSO/95% PBS to a dose of 2.5 mg/kg and also delivered intraperitoneally. All experiments adhered to the guidelines of the Northwestern University Institutional Animal Care and Use Committee (IACUC).

**Antibodies**

Primary antibodies used included: Lamin A/C (Proteintech, 10298-1-AP), Lamin A/C (4C11) Mouse mAb (Cell Signaling Technology, #4777), DNA-PKcs (Abcam, ab70250), DNA-PKcs (E6U3A) Rabbit mAb (Cell Signaling Technology, #38168), Sox2 (D9B8N) Rabbit mAb (Cell Signaling Technology, #23064), Oct-4 Antibody (Cell Signaling Technology, #2750), c-Myc (E5Q6W) Rabbit mAb (Cell Signaling Technology, #18583), CD133 (Novus Biologicals, NB120-16518SS), Beta Actin Polyclonal antibody (Proteintech, 20536-1-AP), and Phospho-Histone H2A.X (Ser139) (20E3) Rabbit mAb (Cell Signaling Technology, #9718). These were applied in Western Blot, Immunofluorescence, and/or Immunoprecipitation protocols

Primary antibodies used included: Lamin A/C (Proteintech, 10298-1-AP), Lamin A/C (4C11) Mouse mAb (Cell Signaling Technology, #4777), DNA-PKcs (Abcam, ab70250), DNA-PKcs (E6U3A) Rabbit mAb (Cell Signaling Technology, #38168), Sox2 (D9B8N) Rabbit mAb (Cell Signaling Technology, #23064), Oct-4 Antibody (Cell Signaling Technology, #2750), c-Myc (E5Q6W) Rabbit mAb (Cell Signaling Technology, #18583), CD133 (Novus Biologicals, NB120-16518SS), Beta Actin Polyclonal antibody (Proteintech, 20536-1-AP), Phospho-Histone H2A.X (Ser139) (20E3) Rabbit mAb (Cell Signaling Technology, #9718), Phospho-ATM (Ser1981) (D6H9) Rabbit mAb (Cell Signaling Technology, #5883), Phospho-ATR (Ser428) Rabbit mAb (Cell Signaling Technology, #2853), Phospho-BRCA1 (Ser1524) Rabbit mAb (Cell Signaling Technology, #9009), Phospho-Chk1 (Ser345) (133D3) Rabbit mAb (Cell Signaling Technology, #2348), Phospho-Chk2 (Thr68) (C13C1) Rabbit mAb (Cell Signaling Technology, #2197), Phospho-p53 (Ser15) Rabbit mAb (Cell Signaling Technology, #9284), PARP1 (46D11) Rabbit mAb (Cell Signaling Technology, #9532), and SOD2 (D9V9C) Rabbit mAb (Cell Signaling Technology, #13194). These were applied in Western blot, immunofluorescence, immunohistochemistry, and/or immunoprecipitation protocols as appropriate.

**Statistical Analysis**

In vitro experiments were conducted in biological triplicate (n=3) with at least one independent replication to ensure reproducibility. For animal studies, a sample size of 5 mice per group (Vehicle, TMZ, KU57788, TMZ+KU57788; N=5/group) was determined based on prior studies and pilot experiments, providing sufficient power (80%) to detect a biologically meaningful effect size at a significance level of 0.05. No samples or animals were excluded from analyses unless technical failures occurred (e.g., unsuccessful tumor engraftment or culture contamination), as per pre-established criteria. All statistical analyses were performed using GraphPad Prism v9.0 and RStudio. Survival outcomes were compared using the Log-Rank test, with treatment effects relative to vehicle control quantified via Cox proportional hazards regression, yielding Hazard Ratios (HR) with 95% Confidence Intervals (CI), visualized in forest plots. For in vitro assays (e.g., cell viability, proliferation), two-sided Student’s t-tests were applied after confirming normality with the Shapiro-Wilk test; non-parametric Wilcoxon tests were used if data were non-normal.Data are presented as mean ± standard deviation (SD). No blinding was performed during data collection or analysis. P-values are reported in the text and figures, with statistical significance defined as p<0.05.

**Mass Spectrometry**

Dr. Weidong Zhou performed mass spectrometry at George Mason University. Each IP sample was mixed with 8 M urea and reduced with 10 mM dithiothreitol at 50 °C for 5 minutes. The mixture was alkylated with 50 mM iodoacetamide at room temperature for 15 minutes and digested with trypsin at 37 °C for 4 hours. The sample was desalted by ZipTip, dried in SpeedVac, then reconstituted with 10 µL of 0.1% formic acid for mass spectrometry (MS) analysis. Liquid chromatography coupled tandem mass spectrometry (LC-MS/MS) experiments were performed on an Exploris 480 (ThermoFisher Scientific, Waltham, MA, USA) equipped with a nanospray EASY-nLC 1200 HPLC system. Peptides were separated using a reversed-phase PepMap RSLC 75 μm i.d. × 15 cm long with 2 μm particle size C18 LC column from ThermoFisher Scientific. The mobile phase consisted of 0.1 % aqueous formic acid (mobile phase A) and 0.1 % formic acid in 80% acetonitrile (mobile phase B). After sample injection, the peptides were eluted by using a linear gradient from 5% to 40% B over 90 minutes and ramping to 100% B for an additional 2 minutes. The flow rate was set at 300 nL/min. The Explores 480 was operated in a data-dependent mode in which one full MS scan (60,000 resolving power) from 300 m/z to 1500 m/z was followed by MS/MS scans in which the most abundant molecular ions were dynamically selected and fragmented by Higher-energy collisional dissociation (HCD) using a collision energy of 27%. “EASY-Internal Calibration,” “Peptide Monoisotopic Precursor Selection,” and “Dynamic Exclusion” (15-sec duration) were enabled, as was the charge state dependency so that only peptide precursors with charge states from +2 to +4 were selected, and fragmented. Tandem mass spectra were searched against the NCBI human database using Proteome Discover v 2.3 from ThermoFisher Scientific. The SEQUEST node parameters were set to use full tryptic cleavage constraints with dynamic methionine oxidation. The mass tolerance for precursor ions was 2 ppm, and mass tolerance for fragment ions was 0.02 Da. A 1% false discovery rate (FDR) was used as a cut-off value for reporting peptide spectrum matches (PSM) from the database search. Data visualization was performed using peptide spectrum matches and GraphPad Prism v9.0.

**Bioinformatic analysis**

Single-cell RNA-seq data from patient-derived xenograft (PDX) glioblastoma (GBM) samples were analyzed using an integrated R pipeline that combined Seurat with several complementary packages. First, the integrated Seurat object was normalized using the SCT assay, and expression levels for LMNA and PRKDC were extracted. Otsu thresholding (implemented via EBImage) was then applied to establish gene-specific cutoffs, classifying cells as “High_Coexpress” when both genes exceeded their respective thresholds. Histograms, donut plots, and UMAPs were generated following PCA-based dimensionality reduction and clustering (using the top 10 principal components at a resolution of 0.5) to visualize expression distributions. Differential expression analysis, carried out with the Wilcoxon test, compared “High_Coexpress” cells to all others. Key markers were highlighted in volcano plots and heatmaps when they met the criteria of an adjusted p-value < 0.05 and an absolute log₂ fold-change > 0.25. For functional interpretation, differentially expressed genes were mapped to Entrez IDs and analyzed via overrepresentation analysis (ORA) and gene set enrichment analysis (GSEA) for Gene Ontology (BP, MF, CC) and MSigDB C2 CP:PID gene sets using clusterProfiler. Custom dot plots summarized the enrichment results, and gene signature module scores were computed using Seurat’s AddModuleScore function. These scores were visualized with additional dot plots to compare average signature expression across different cell groups and experimental conditions. In addition to these analyses, spatial plots were generated to visualize gene expression in a spatial context.

Subsequently, the GBmap integrated object was loaded and verified for the RNA assay.[11] Ensembl IDs were mapped to gene symbols using org.Hs.eg.db, and multiple UMAP plots were generated to display the resulting annotations. Finally, cells expressing LMNA and PRKDC, as well as those co-expressing both genes, were highlighted, and additional dot plots were produced to summarize co-expression frequencies across the various annotations.

***Generation of knockdown and overexpression lines***

shRNA knockdown (KD) plasmids were obtained from GeneCopoeia as glycerol stocks, cultured overnight in LB-Ampicillin liquid media, and extracted from bacteria using the Qiagen Plasmid Midi Kit. The overexpression (OE) plasmid was obtained from Addgene and prepared similarly. HEK293 cells were transfected with scramble control, 3 different shRNA KD clones, and an OE plasmid along with 2^nd^ generation gag-pol and packaging plasmids in a 6:6:2 ratio using X-tremeGENE 360 Transfection Reagent and OptiMEM media. Cells were incubated for 72-hours at which point a supernatant containing lentiviral particles was collected from each of the plates and used to infect U251, GBM6, GBM6R, and GBM43 cells via a spinfection protocol.

***Immunofluorescence***

For *ex vivo* immunofluorescence, the brains of nude mice from the previously described animal studies were harvested after the animals succumbed to tumor burden and frozen at -80°C in optimal cutting temperature (OCT) compound. Tissue was sectioned using a cryostat in 8 micron sections and adhered to glass microscope slides. Sections were blocked for 2 hours at room temperature, incubated overnight at 4°C in primary antibody solutions, and incubated for 2 hours at room temperature in secondary antibody solutions. Sections were mounted using ProLong™ Gold Antifade reagent with DAPI mounting media and imaged using a Leica DMi8 microscope.

***Immunoprecipitation***

Following the completion of treatments, cells were trypsinized, washed 1X in PBS, and resuspended in mPER buffer. Reactions were set up with 100ug of protein and 2uL of antibody per tube. Following overnight incubation at 4°C, reactions were precipitated for 2 hours at room temperature with gentle rotation, washed 3X with mPER buffer, and eluted with 2X reducing SDS buffer at 55°C for 10 minutes. Samples were finally boiled for 7 minutes at 95°C and used to run a western blot.

***Extreme limiting dilution assay***

Following completion of treatments, cells were trypsinized and diluted in neurobasal medium (NBM; 1% antibiotic/antimycotic, 1% glutamine, 2% N2-supplement, 1% B27-supplement, 0.02% EGF, and 0.02% FGF) to a final concentration of 1000 cells/mL. Cells were then plated in a 96-well as 12-well replicates at 8 different concentrations from 3 cells/well to 200 cells/well. Cells were incubated for 1 week at which point wells were assessed for neurosphere formation. All analysis was done using online software created by Yifang Hu (<http://bioinf.wehi.edu.au/software/elda/index.html>).


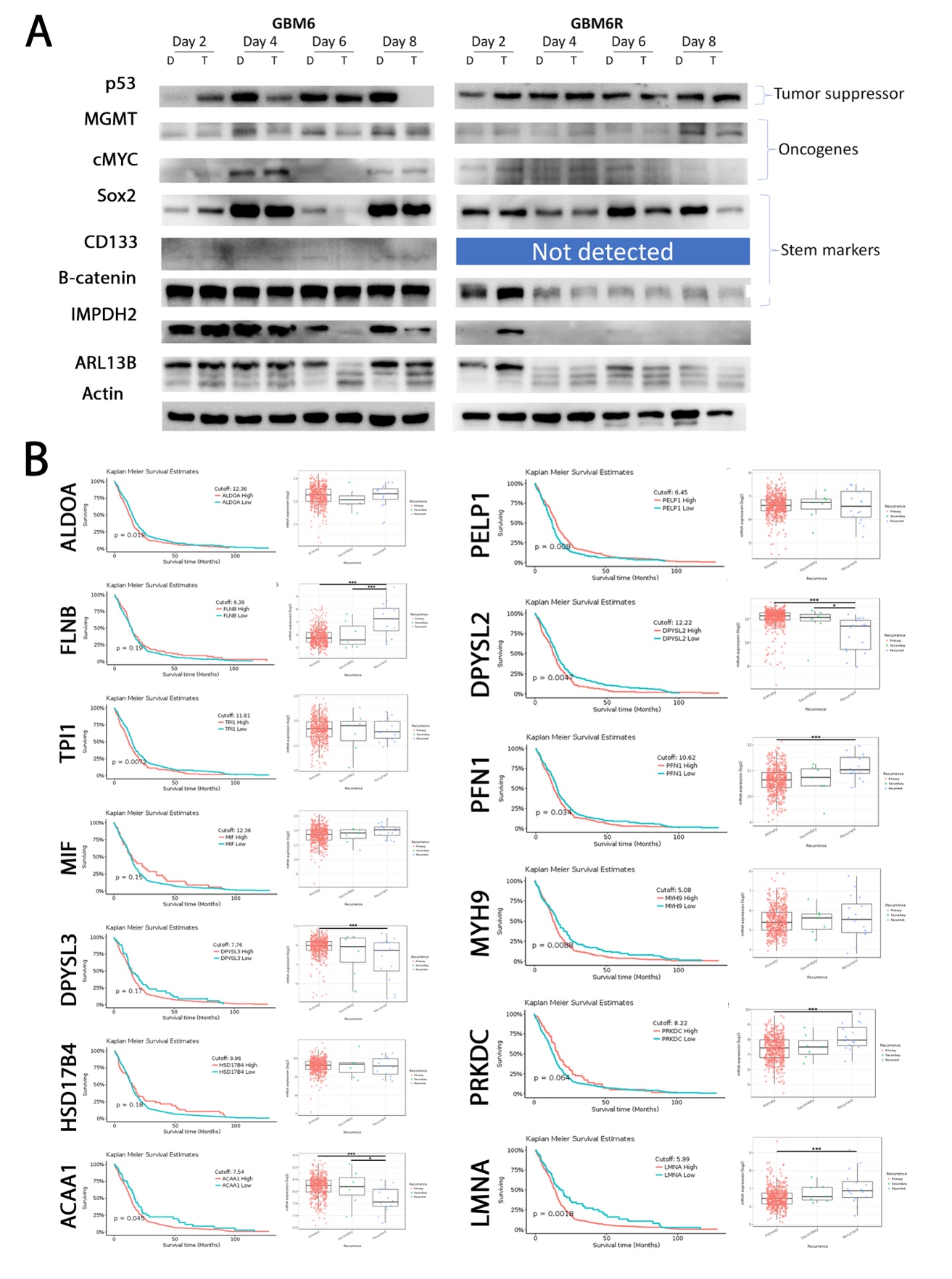
 **Supplementary Figure 1:**

**Supplementary Figure 1: Prognostic significance and expression patterns of candidate genes in glioblastoma.** **A.** Immunoblot analysis of GBM6 and GBM6R cells collected at Day 2, 4, 6, and 8 following DMSO or TMZ treatment. Blots were probed for key tumor suppressors, oncogenes, and stem cell–associated markers to assess dynamic changes over time. **B.** Kaplan–Meier survival analyses from the GlioVis TCGA_GBM dataset comparing overall survival in patients with high versus low expression levels of the top genes identified by IP-MS analysis:  ALDOA,  PELP1,  FLNB,  DPYSL2,  TPI1,  PFN1, MIF,  MYH9,  DPYSL3,  PRKDC,  HSD17B4,  LMNA, and  ACAA1. Higher or lower gene expression was stratified based on median mRNA levels, and survival significance was calculated accordingly. Boxplots showing mRNA expression levels of the same genes across primary, secondary, and recurrent glioblastoma subtypes from TCGA_GBM data in GlioVis. Statistical comparisons were performed using Tukey’s HSD test to evaluate differences in gene expression across tumor progression stages.


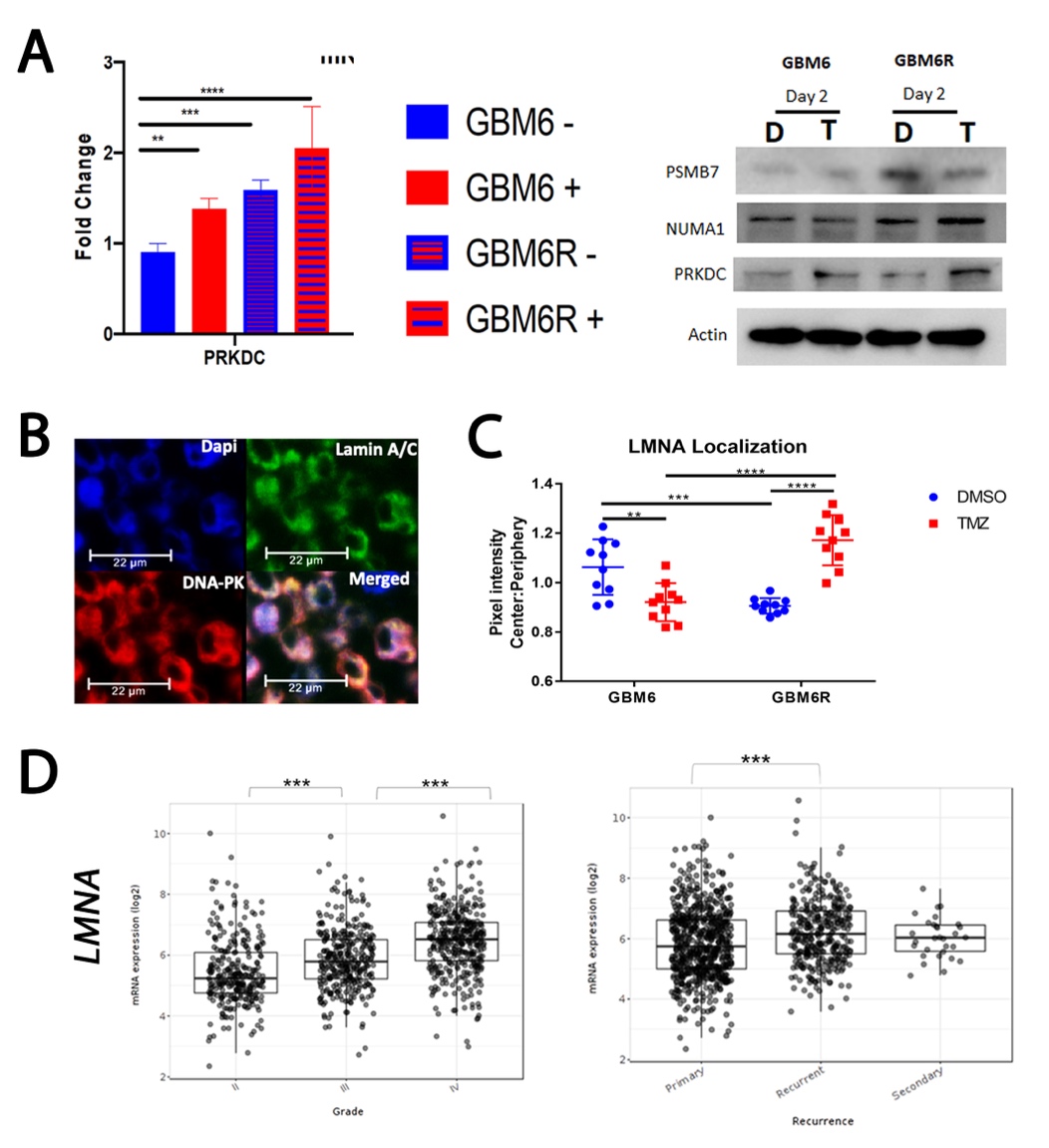
**Supplementary Figure 2:**

**Supplementary Figure 2: LMNA localization, expression dynamics, and clinical relevance in glioma. A.** Left: Quantitative PCR analysis of PRKDC expression in GBM6 and GBM6R cells treated with DMSO or TMZ (50 µM), showing elevated baseline and treatment-induced expression in GBM6R. Right: Immunoblot validation of the top three candidate proteins identified through mass spectrometry in GBM6R versus GBM6 cells. **B.** Immunohistochemical (IHC) staining of patient-derived GBM43 tumor tissue showing nuclear DAPI (blue), Lamin A/C (green), and DNA-PK (red). Co-localization highlights nuclear LMNA and PRKDC expression. **C.** Quantification of LMNA subcellular localization in GBM6 and GBM6R cells treated with DMSO or TMZ (50 µM), showing differential localization patterns in response to treatment. **D.** Left: mRNA expression analysis of LMNA in the GlioVis CGGA dataset reveals significant increases across tumor grades—Grade II vs. Grade III and Grade III vs. Grade IV. Right: LMNA mRNA levels are also significantly higher in recurrent tumors compared to primary tumors. Statistical significance was determined using Tukey’s HSD test.

**Supplementary Figure 3:**


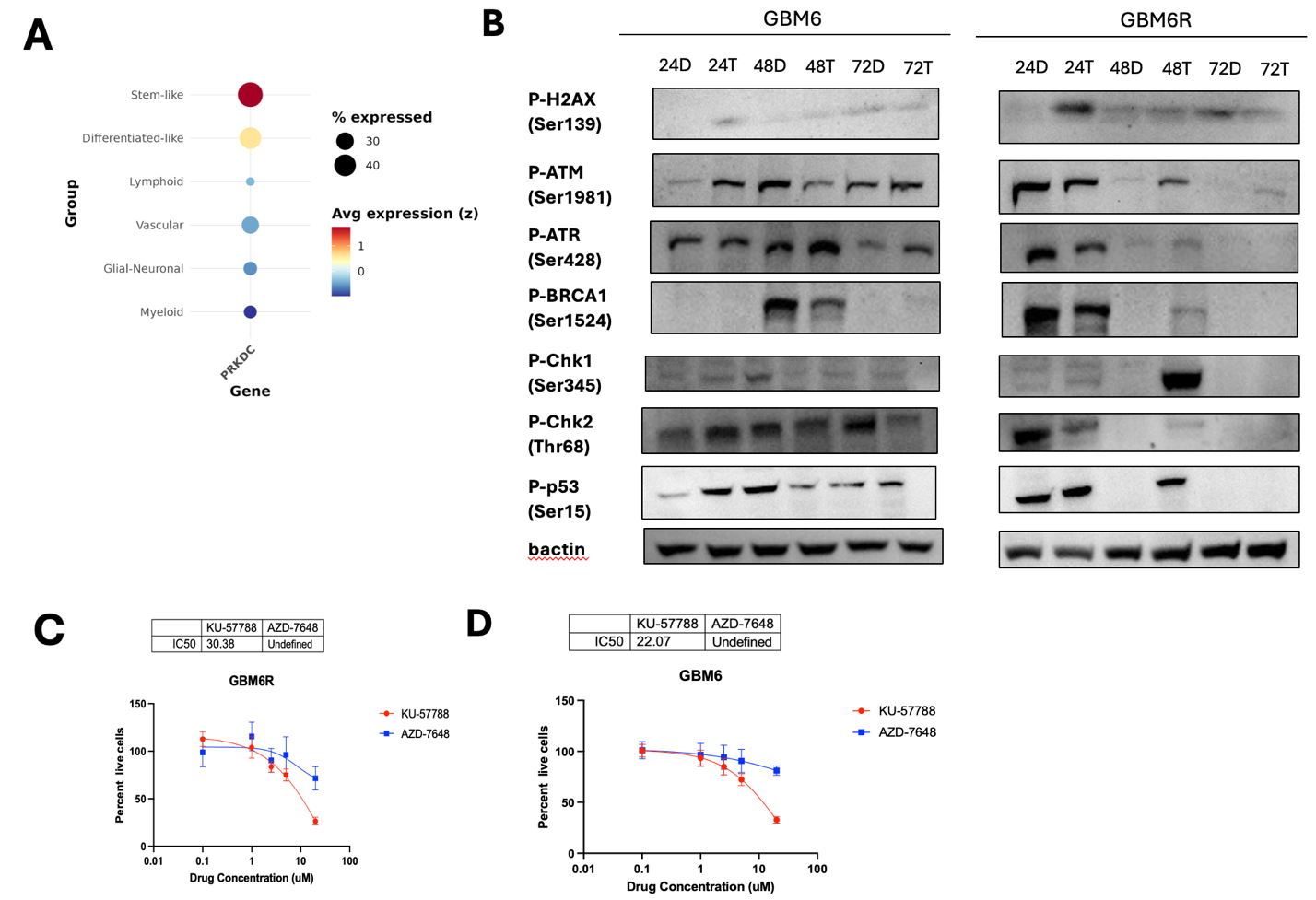


**Supplementary Figure 3: PRKDC dependency and DNA damage response in GBM. A.** Single-cell transcriptomic analysis (GBmap) demonstrating PRKDC has higher expression in stem-like and differentiated-like subpopulations compared to other cellular states. **B.** Time-course western blots of γH2AX and a DNA damage response (DDR) panel (p-ATM, p-ATR, p-BRCA1, p-Chk1, p-Chk2, p-p53) in GBM6 and GBM6R cells following vehicle (D) or TMZ (T) treatment, showing decreased damage signaling in GBM6R. **C-D.** Cell viability assays in GBM6 and GBM 6R cells treated with KU57788 or AZD7648. KU57788 reached IC₅₀ values of ~22-30 µM, while AZD7648 does not reach IC₅₀.

**
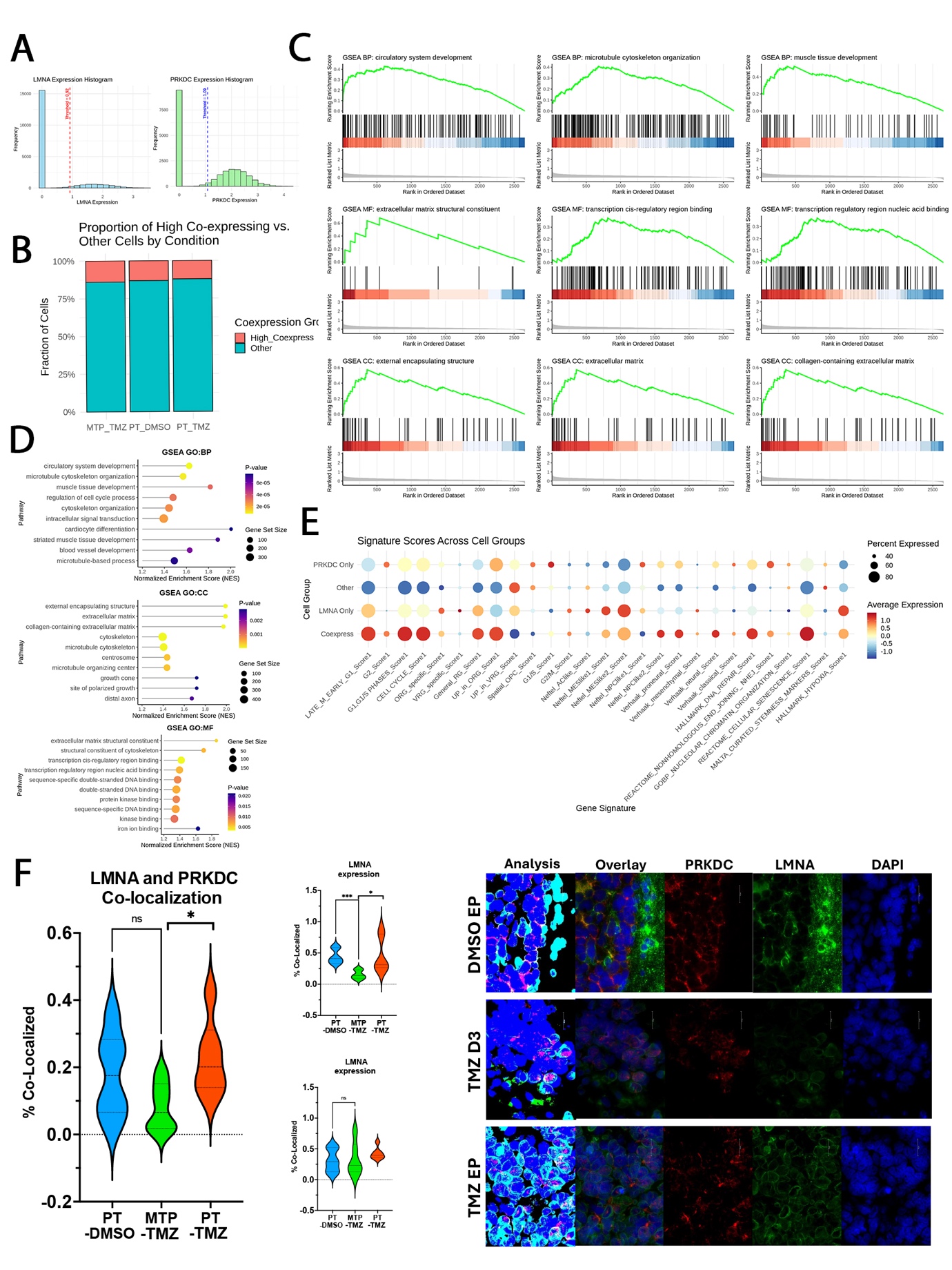
Supplementary Figure 4:**

**Supplementary Figure 4:** **LMNA and PRKDC co-expression defines transcriptionally distinct subpopulations with enriched biological pathways.** **A.** Top: Otsu threshold histograms displaying the LMNA and PRKDC expression distributions in single-cell RNA-seq data. Dashed vertical lines indicate the Otsu-derived cutoffs used to stratify cells into high-expression groups. Bottom: A stacked bar chart shows the relative abundance of high LMNA/PRKDC co-expressing cells versus all other cells across experimental conditions. **B.** Gene set enrichment analysis (GSEA) plots depicting the top nine pathways significantly enriched in high co-expressing cells. **C.** Gene set enrichment dot plots illustrating the top 10 enriched pathways across Gene Ontology Biological Process (GO:BP), Molecular Function (GO:MF), and Cellular Component (GO:CC) categories. Dot size reflects gene set size; color intensity indicates statistical significance based on normalized enrichment scores (p-values [specify if adjusted or unadjusted]). **D.** Module score dot plot showing average gene signature scores across defined cell groups, highlighting pathway activation in high co-expressing populations. **E.** Immunohistochemistry (IHC) images comparing LMNA and co-localized PRKDC/LMNA expression across treatment conditions. LMNA and co-expression are significantly increased in PT-TMZ versus MTP-TMZ samples, while LMNA levels are decreased in MTP-TMZ relative to PT-DMSO.

**Supplementary Figure 5:**


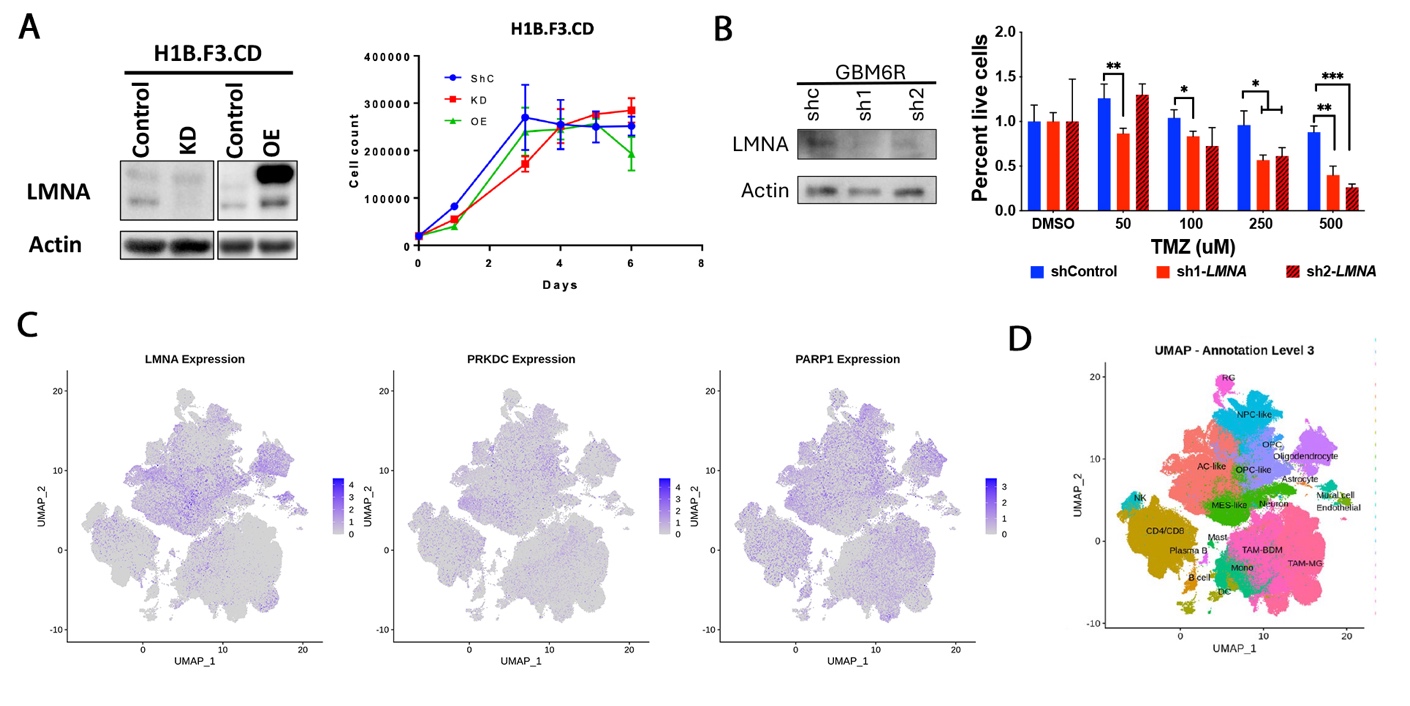


**Supplementary Figure 5:** **Functional manipulation and expression profiling of LMNA in normal and malignant neural cell contexts.** **A.** Left: Immunoblot confirming LMNA knockdown (KD) and overexpression (OE) in the immortalized normal human neural stem cell line HB1.F3.CD. Right: Cell viability assay showing the impact of LMNA KD and OE on neural stem cell response to TMZ. **B.** Left: Immunoblot validation of two independent LMNA shRNAs (sh1 and sh2) in GBM6R cells. Right: Dose–response curve showing TMZ sensitivity in GBM6R cells infected with LMNA sh1 and sh2 compared to scrambled control. **C.** Single-cell RNA-seq expression profiles of LMNA, PRKDC, and PARP1 across GBmap-defined cell populations. **D.** GBmap legend outlining cell type annotations used in the dataset analysis presented in (C).


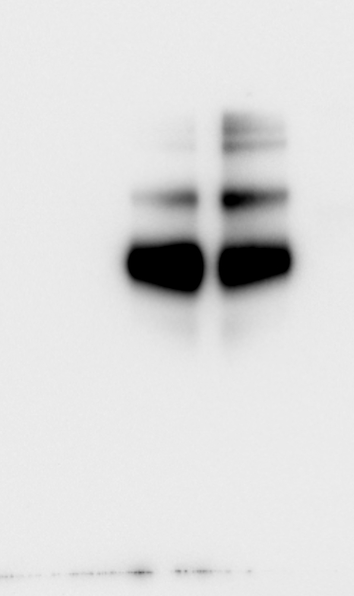

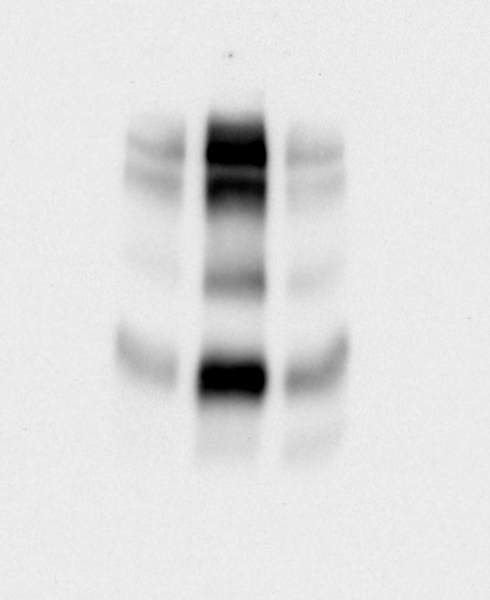

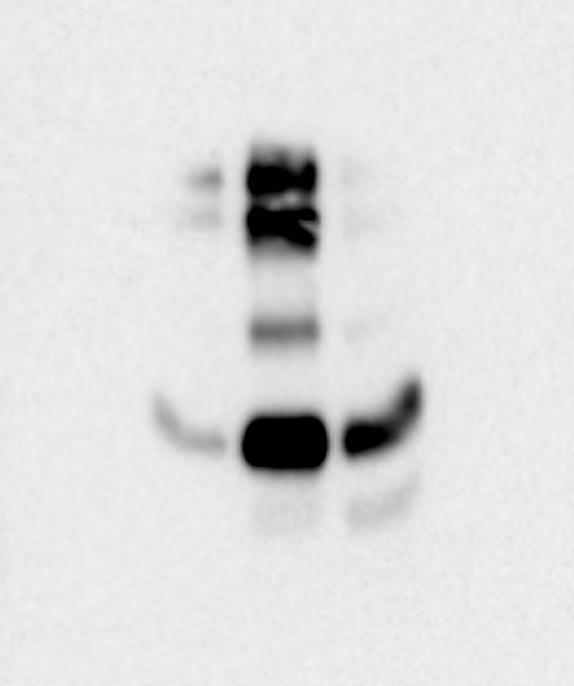
Original data:

PARP1

pPRKDC

LMNA


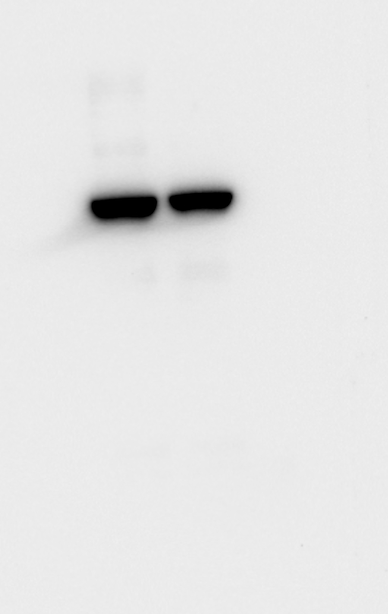

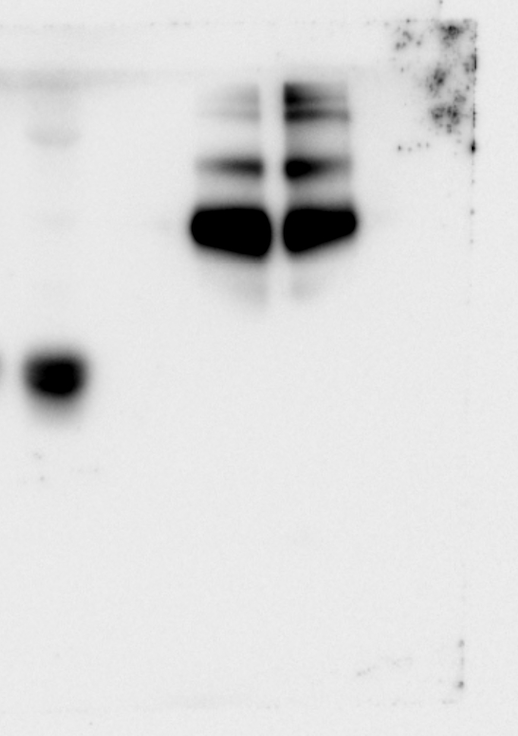

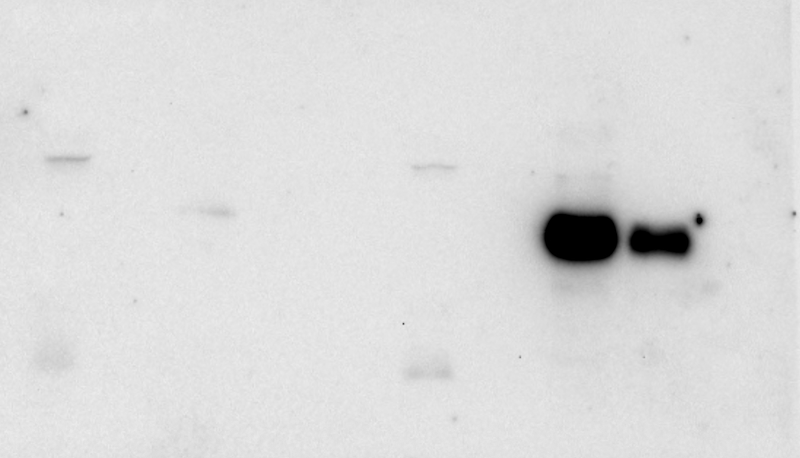

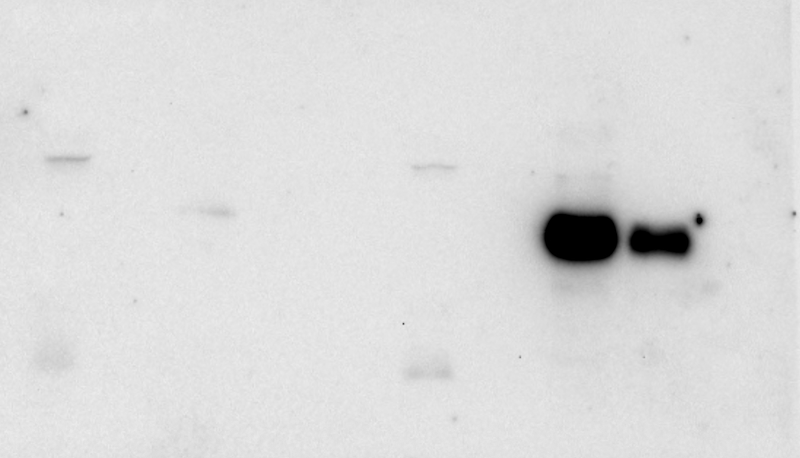

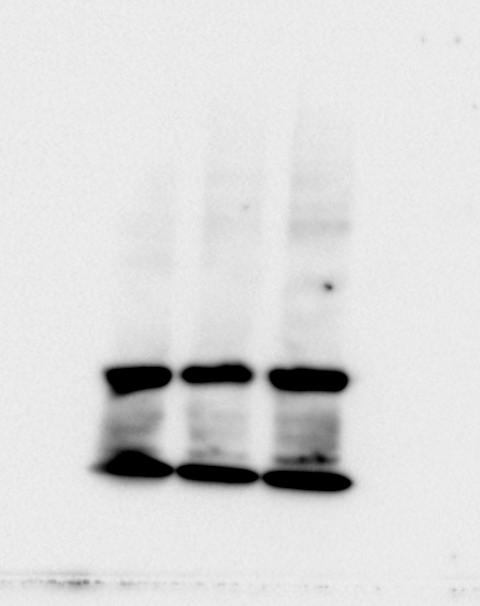

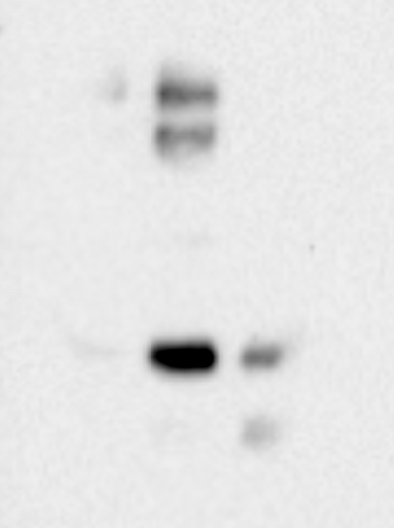

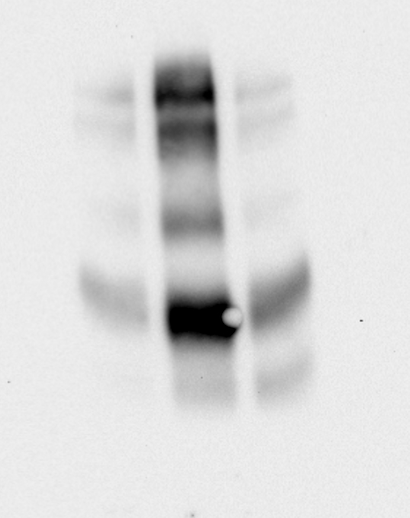


PRKDC

LMNA

Input

SOD2

Input

PRKDC

PARP1


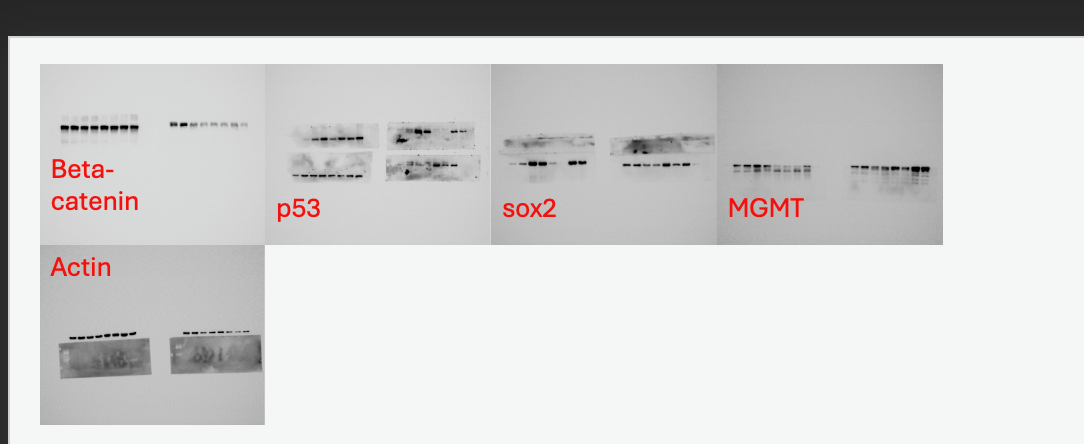


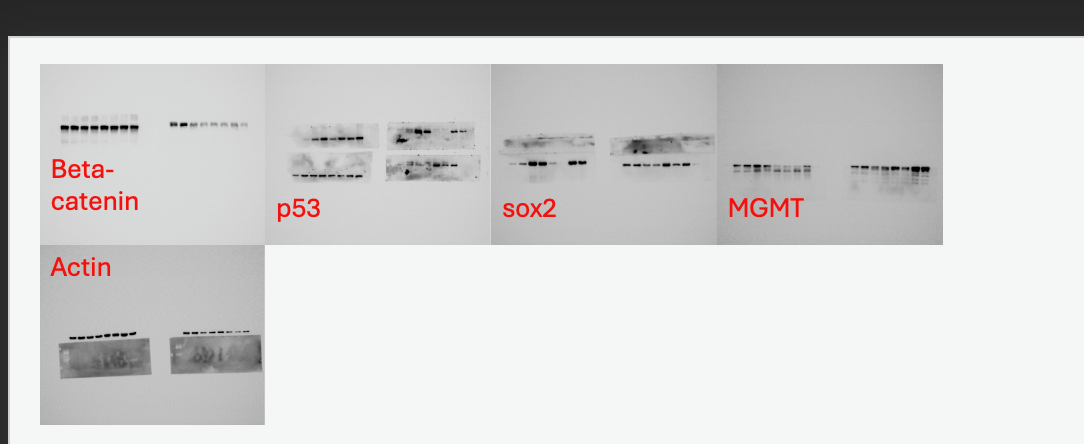


**
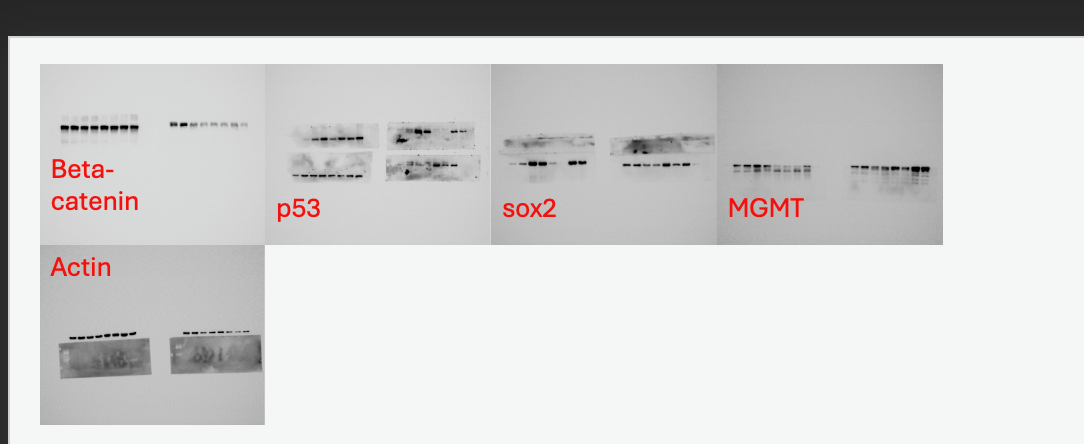
**
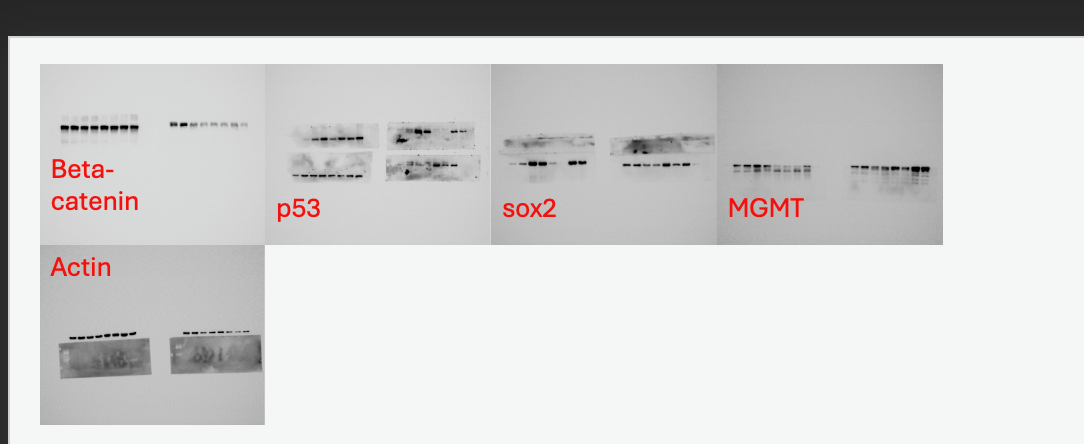
**
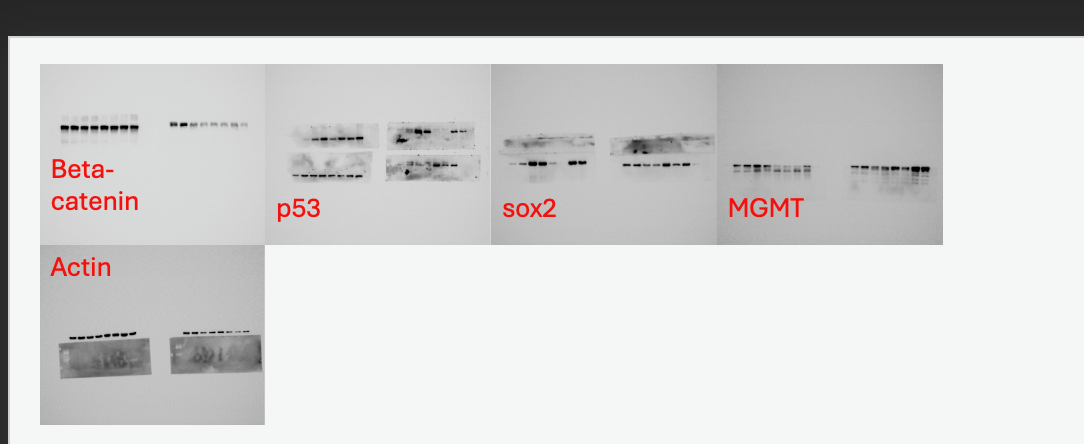
**

**
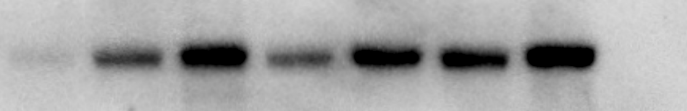

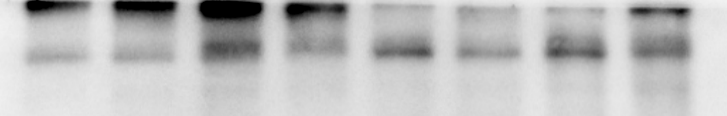

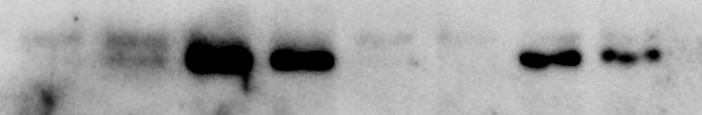

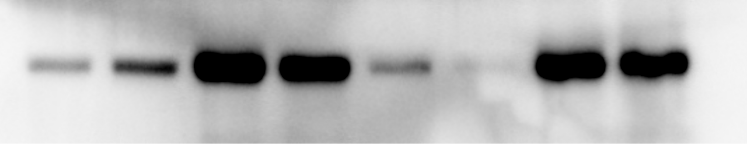

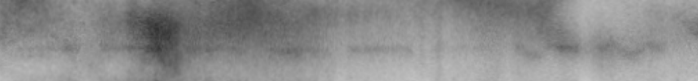

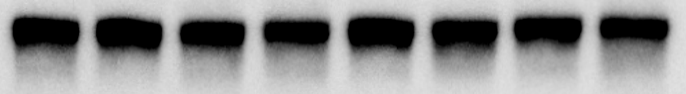

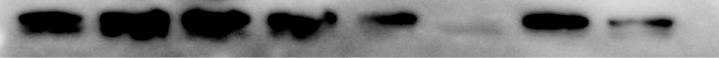

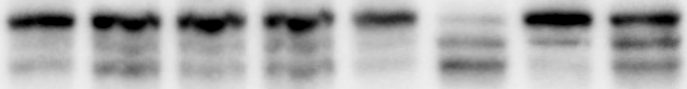

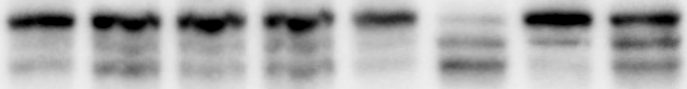

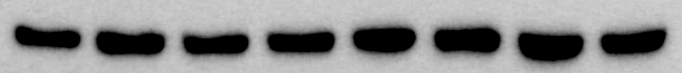
**

D

T

D

T

D

T

D

T

Day 2

Day 4

Day 6

Day 8

p53

MGMT

EN2

Sox2

CD133

B-catenin

IMPDH2

ARL13B

ARL13B

Actin

SF1A

**
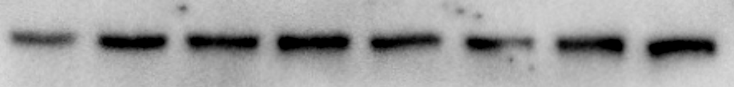

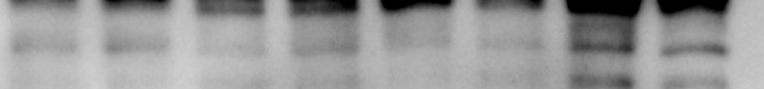

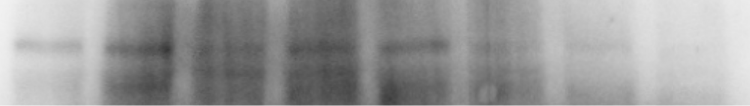

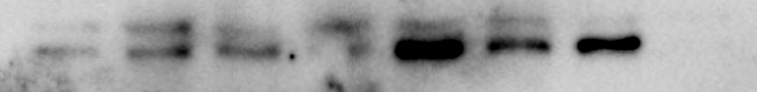

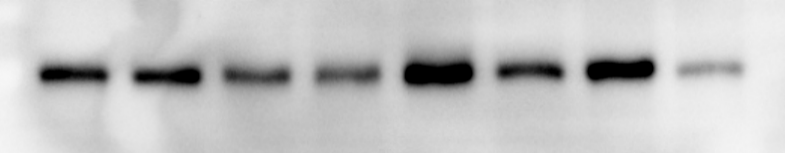

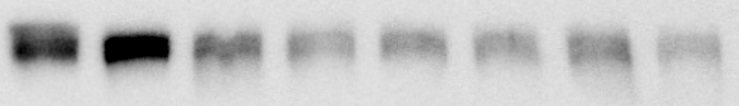

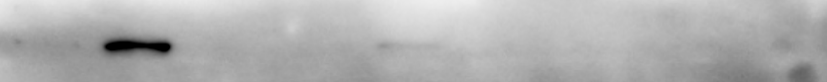

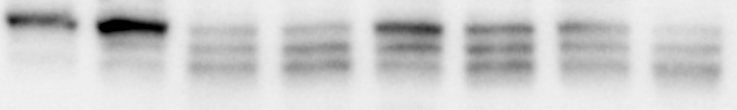

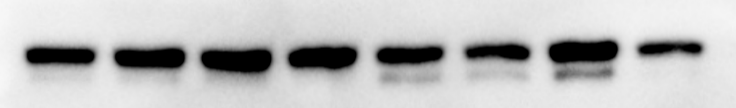
**

p53

MGMT

cMYC

EN2

Sox2

B-catenin

IMPDH2

ARL13B

Actin


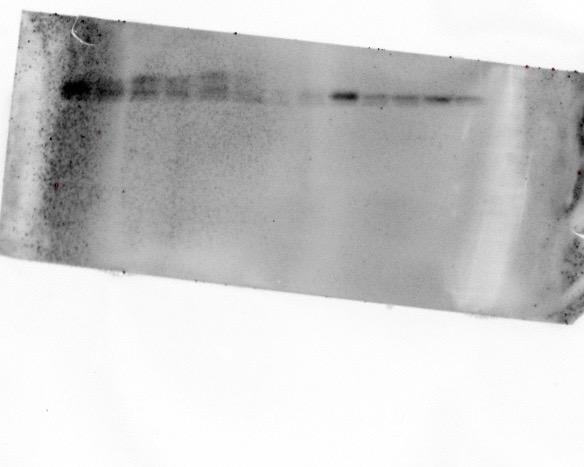

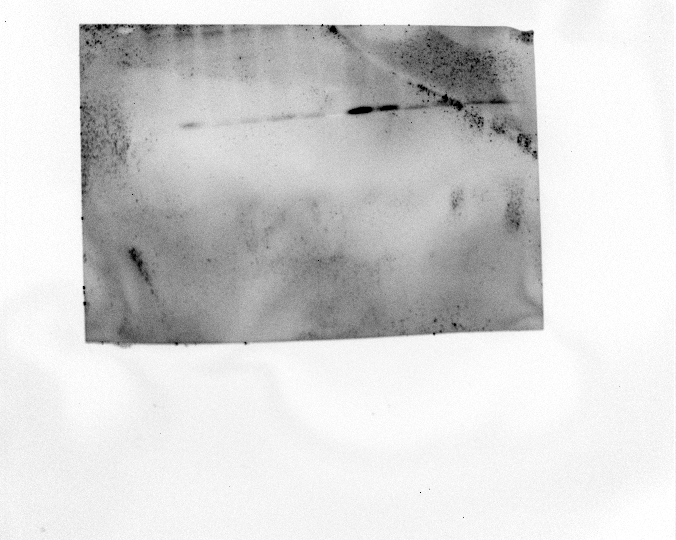


**P-H2AX**

**(Ser139)**


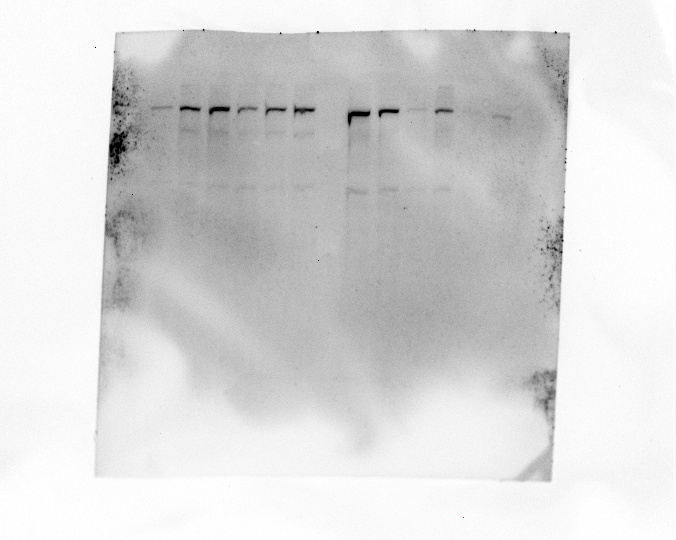

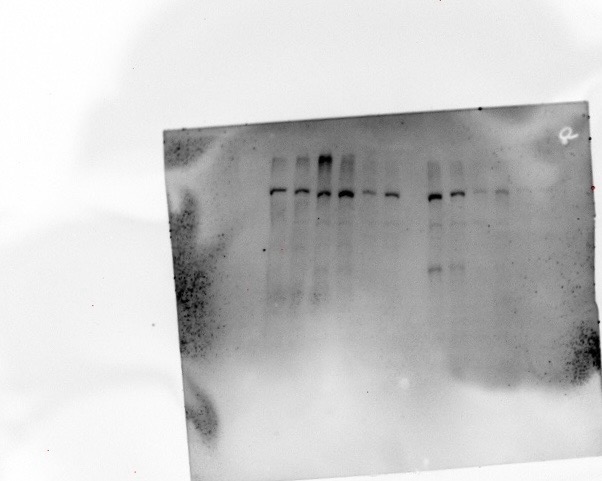

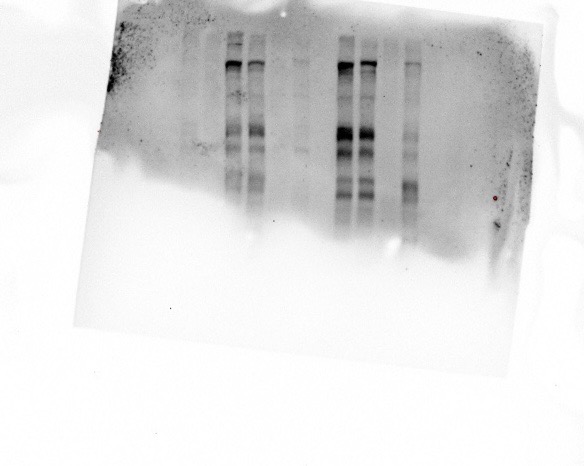


**P-ATR**

**(Ser428)**

**P-BRCA1**

**(Ser1524)**

**P-ATM**

**(Ser1981)**


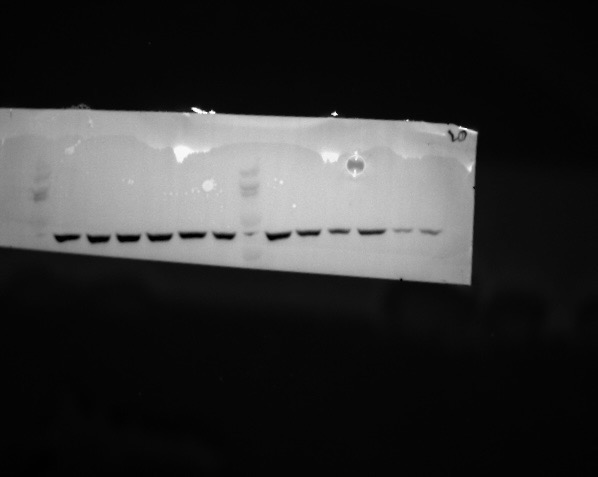

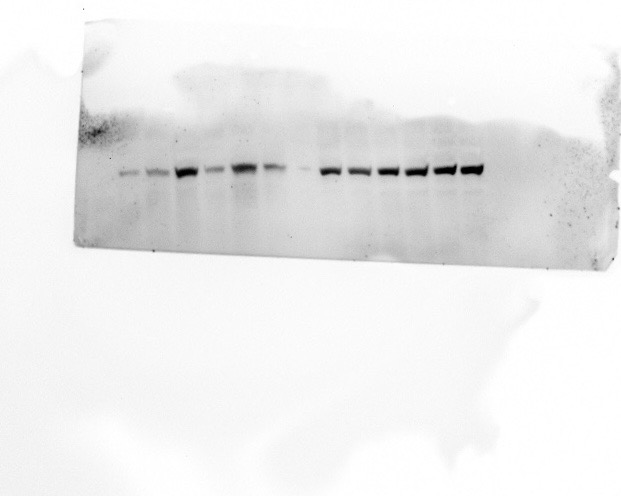

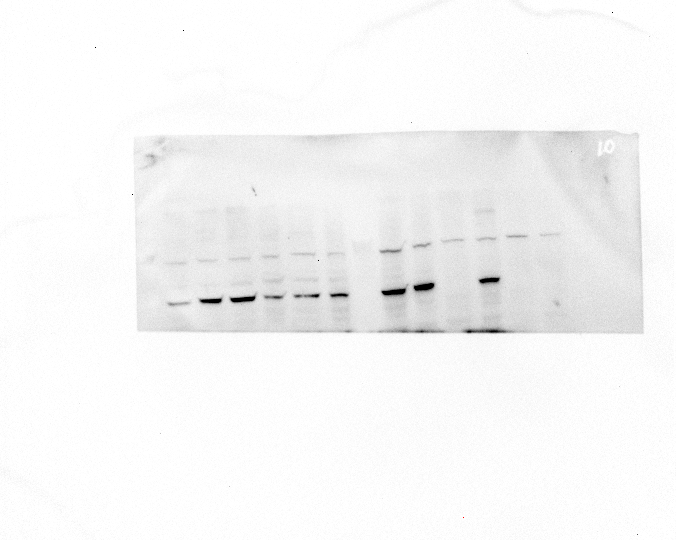

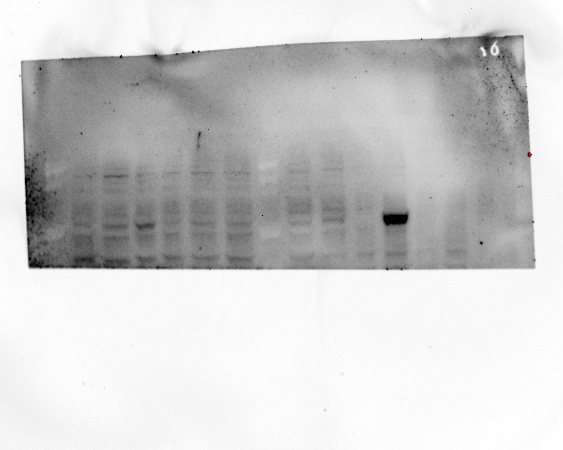

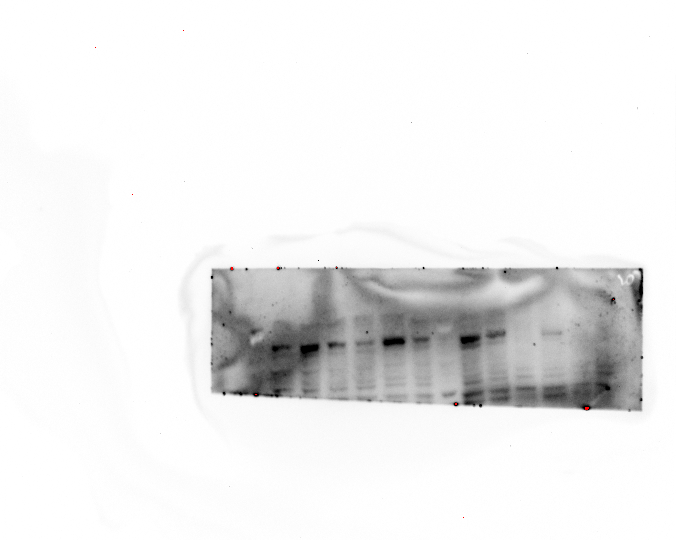

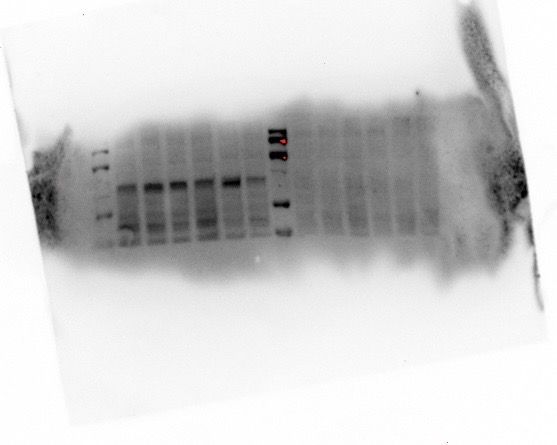


**bactin**

**P-p53**

**(Ser15)**

**P-Chk2**

**(Thr68)**

**P-Chk1**

**(Ser345)**
